# Transformer‐Based Multitask Framework Integrating Habitat and Deep Learning for Predicting Early Disease Control and Survival in Immunotherapy‐Treated Hepatocellular Carcinoma

**DOI:** 10.1002/advs.78005

**Published:** 2026-09-27

**Authors:** Xiaona Fu, Yusheng Guo, Bingxin Gong, Jie Lou, Ning Wang, Xuejun Chen, Hongtao Hu, Rundong Wang, Xiaofang Guo, Shanmei Li, Yangyang Xie, Shichao Long, Mengsi Li, Shubo Pan, Wen Shen, Zhaogang Zhang, Zifang Song, Pengchen Liang, Xiaoyun Liang, Peng Sun, Guilin Zhang, Shanshan Jiang, Jinxiang Zhang, Lian Yang

**Affiliations:** ^1^ Department of Radiology Union Hospital Tongji Medical College Huazhong University of Science and Technology Wuhan China; ^2^ Hubei Provincial Clinical Research Center for Precision Radiology & Interventional Medicine Wuhan China; ^3^ Hubei Key Laboratory of Molecular Imaging Wuhan China; ^4^ Department of Radiology The Affiliated Cancer Hospital of Zhengzhou University & Henan Cancer Hospital Zhengzhou China; ^5^ Department of General Surgery The First Affiliated Hospital of USTC Division of Life Science and Medicine University of Science and Technology of China Hefei China; ^6^ Department of Radiology Hubei Cancer Hospital Tongji Medical College Huazhong University of Science and Technology Wuhan China; ^7^ Zhejiang Key Laboratory of Multi‐omics Precision Diagnosis and Treatment of Liver Diseases Department of General Surgery Sir Run Run Shaw Hospital Zhejiang University School of Medicine Hangzhou China; ^8^ Department of Radiology Xiangya Hospital Central South University Changsha China; ^9^ Department of Hepatobiliary and Pancreatic Surgery The Second Affiliated Hospital of Anhui Medical University Hefei China; ^10^ Department of Radiology Tianjin First Central Hospital Tianjin Institute of Imaging Medicine School of Medicine Nankai University Tianjin China; ^11^ Department of Hepatobiliary Surgery Union Hospital Tongji Medical College Huazhong University of Science and Technology Wuhan China; ^12^ Institute of Research and Clinical Innovations Neusoft Medical Systems Co. Ltd. Shanghai China; ^13^ Institute of Research and Clinical Innovations Neusoft Medical Systems Co. Ltd. Shenyang China; ^14^ Department of Emergency Surgery Union Hospital Tongji Medical College Huazhong University of Science and Technology Wuhan China

**Keywords:** deep learning, hepatocellular carcinoma, immunotherapy, prognosis, treatment outcome

## Abstract

Hepatocellular carcinoma (HCC) patients show heterogeneous responses to immune checkpoint inhibitors (ICIs). This study developed ECOS‐Net, a transformer‐based multitask network integrating CT‐derived habitat and 2.5‐dimensional (2.5D) deep learning features for simultaneously predicting early disease control (DC) and overall survival (OS). Of 1,234 patients with HCC enrolled from eight institutions and public databases, 832 ICI‐treated patients were used for model development. ECOS‐Net fused features using multi‐head attention and generated early DC probabilities and OS risk scores. ECOS‐DC achieved AUCs of 0.836, 0.822, and 0.817 in training, internal validation, and external test sets, outperforming clinical models (all *p* values < 0.05). ECOS‐OS yielded C‐indices of 0.730, 0.722, and 0.720, respectively. Integrated models also showed favorable external performance (early DC AUC: 0.825; OS C‐index: 0.741). Patients with higher ECOS‐DC probabilities had a higher likelihood of early DC, whereas those with higher ECOS‐OS risk had shorter OS, with directionally consistent associations across most subgroups. Exploratory biological analyses suggested that the higher ECOS‐DC probability and lower ECOS‐OS risk groups were associated with immune‐active tumor microenvironment features. Therefore, ECOS‐Net shows potential as a non‐invasive imaging‐based risk stratification framework for simultaneously predicting early DC and OS in ICI‐treated HCC patients.

## Graphical abstract

This study developed ECOS‐Net, a transformer‐based multitask framework integrating CT‐derived habitat and 2.5D deep learning features to simultaneously predict early disease control and overall survival in hepatocellular carcinoma patients treated with immune checkpoint inhibitors. Multicenter retrospective results indicated its potential as a non‐invasive imaging‐based risk stratification framework, while exploratory multi‐omics, pathology, and single‐cell RNA analyses suggested immune‐related biological correlates.

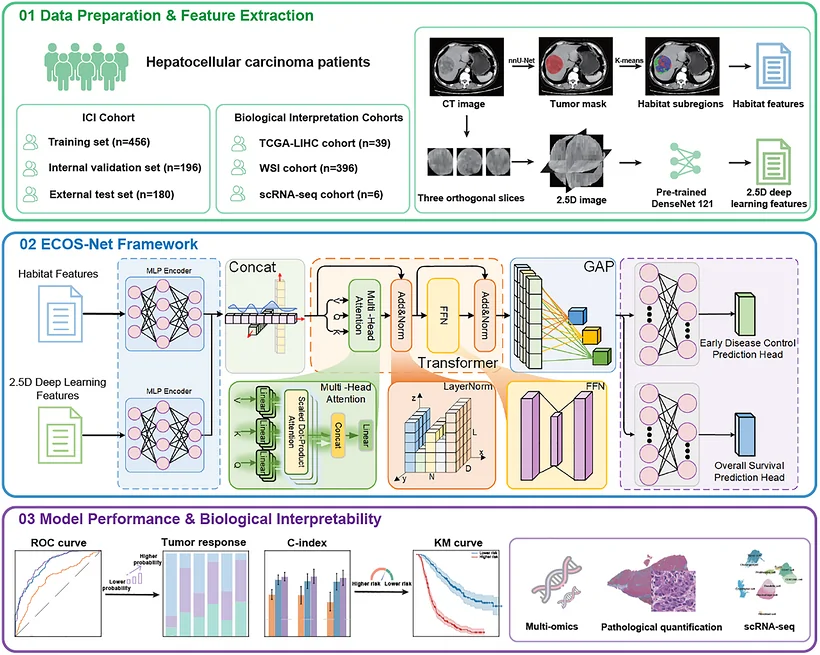

## 1 Introduction

Hepatocellular carcinoma (HCC) remains the most common type of primary liver cancer globally [1]. Although the advent of immune checkpoint inhibitors (ICIs) has improved survival outcomes for patients and reshaped the therapeutic landscape for advanced HCC, clinical responses remain highly heterogeneous [2, 3]. Intratumor heterogeneity (ITH), which encompasses spatial variations in genomic, phenotypic, and microenvironmental features within a tumor, is a key driver of both primary and acquired resistance to ICIs in HCC [4, 5]. Specifically, spatial differences in immune infiltration, antigen presentation, and immunosuppressive states within the tumor influence early tumor response after ICI treatment and thereby affect early disease control (DC) [3]. Meanwhile, ITH further affects disease progression and survival outcomes through clonal evolution, immune escape, and the emergence of resistant phenotypes [6, 7]. In clinical practice, early DC indicates initial therapeutic benefit and is closely linked to treatment continuation, whereas overall survival (OS) remains the definitive endpoint for evaluating long‐term immunotherapy benefit. Together, these complementary dimensions of immunotherapy benefit underscore the need for biomarkers capable of predicting both early response and long‐term survival to support individualized treatment decision‐making.

Conventional histopathological and molecular biomarkers, such as programmed death‐ligand 1 (PD‐L1) expression, tumor mutational burden (TMB), and microsatellite instability (MSI), have limited utility in HCC due to low positive detection rates, suboptimal reproducibility, and restricted clinical generalizability [8, 9]. Moreover, these biomarkers are often derived from localized tissue sampling and therefore incompletely capture the complexity of ITH. In contrast, computed tomography (CT) images provide comprehensive lesion information and offer a non‐invasive approach to extracting ITH‐related imaging features. Radiomics enables quantitative characterization of tumor morphology and texture from routine imaging and has shown potential for predicting immunotherapy response and survival in HCC [10, 11]. However, most radiomic models relied on global tumor‐level features and may not fully reflect ITH [12]. Habitat analysis addresses this limitation by using voxel‐level, multidimensional features and unsupervised clustering to delineate functionally distinct subregions, thereby capturing spatial heterogeneity more precisely [13]. Shen et al. [14] reported that a CT‐based habitat model effectively predicted treatment response and survival in HCC patients receiving combination regimens including locoregional therapy and ICIs. In addition, Chen et al. [15] showed that a CT‐based habitat model outperformed a conventional radiomics model in predicting OS, further highlighting the unique advantage of habitat analysis for imaging‐based characterization of ITH.

In recent years, deep learning (DL) has enabled the automatic extraction of high‐level imaging features directly from medical images, providing a powerful approach for characterizing ITH. Xia et al. [16] developed a convolutional recurrent neural network model using representative two‐dimensional (2D) CT images to generate a radiological score for predicting survival in advanced HCC patients receiving immunotherapy. Lin et al. [17] further constructed a multimodal model integrating radiomics, DL, and clinical data, achieving satisfactory performance in predicting the durable clinical benefit of ICI‐based treatment in potentially convertible HCC patients. Regarding feature construction strategies, the 2.5‐dimensional (2.5D) approach incorporates multiplanar image inputs, thereby introducing spatial context while maintaining manageable model complexity and training stability. For multi‑scale information fusion, the transformer‑based framework leverages a self‑attention mechanism to integrate imaging features from different scales within a unified architecture [18]. Chen et al. [19] used a pre‐trained ResNet50 to extract 2.5D features and subsequently incorporated a transformer model to predict treatment response in unresectable HCC patients receiving targeted therapy combined with immunotherapy. These studies highlighted the important role of DL in predictive modeling, from efficient feature extraction to multi‐scale information fusion. Nevertheless, most studies focused on a single clinical endpoint, such as short‐term response or survival, and did not comprehensively evaluate clinical benefit across different time scales. Some models relied mainly on a single level of imaging information, leaving the complementary value of different imaging features insufficiently explored. In addition, many studies lacked adequate multicenter external validation, which limited the assessment of generalizability. Finally, the relationship between imaging‐based predictions and underlying tumor biology remained insufficiently clarified, resulting in limited biological interpretability.

Habitat features and 2.5D DL features provide complementary imaging perspectives for characterizing ITH. Habitat features explicitly describe spatial heterogeneity across intratumoral subregions, whereas 2.5D DL features automatically capture high‐level imaging information directly from original CT images that is not fully characterized by handcrafted features. The integration of these two types of features has the potential to provide a more comprehensive characterization of ITH and improve the prediction of treatment outcomes. Nevertheless, simple concatenation or conventional machine‐learning models may not fully model their interdependencies. The transformer‐based self‐attention mechanism can learn relationships among imaging features at different scales [18, 20], thereby facilitating the integration of habitat and 2.5D DL features. In addition, early DC and OS likely share certain ITH‐related imaging information but represent distinct clinical endpoints. A multitask framework can therefore leverage shared information from both endpoints while retaining endpoint‐specific predictions. To our knowledge, the integration of CT‐derived habitat features and 2.5D DL features within a multitask framework for jointly predicting early DC and long‐term OS in HCC patients receiving ICIs remains insufficiently explored. Therefore, we developed the early control and overall survival network (ECOS‐Net), a transformer‑based fusion framework for concurrently predicting early DC and OS. To improve interpretability, we further explored the potential biological associations of ECOS‐Net predictions with multi‐omics data, pathological features, and single‐cell RNA sequencing (scRNA‐seq) data.

## 2 Experimental Section

### 2.1 Patients and Study Design

A total of 1234 patients with HCC were ultimately enrolled from eight independent institutions and public databases. Publicly available HCC patient data were obtained from The Cancer Genome Atlas (TCGA, https://xena.ucsc.edu), with corresponding imaging data obtained from The Cancer Imaging Archive (TCIA, https://www.cancerimagingarchive.net). Moreover, the scRNA‐seq data used in this study were derived from a subset of patients enrolled in the prospective observational cohort study “Radiogenomics‐Liver”, which was established to investigate HCC imaging features from the perspective of scRNA‐seq. This multicenter study was approved by Union Hospital, Tongji Medical College, Huazhong University of Science and Technology (WHUH), Henan Cancer Hospital (HCH), Anhui Provincial Hospital (APH), Xiangya Hospital, Central South University (XYH), Hubei Cancer Hospital (HBCH), The Second Affiliated Hospital of Anhui Medical University (SAHMU), Tianjin First Central Hospital (TFCH), and Sir Run Run Shaw Hospital, Zhejiang University School of Medicine (SRRSH). Due to the retrospective and anonymous analysis of the clinical data, the Ethics Committees waived the requirement for written informed consent from participants or their legal guardians/next of kin. In addition, the prospective sequencing cohort “Radiogenomics‐Liver” was registered at ClinicalTrials.gov (NCT06536998) and approved by the WHUH Ethics Committee, with written informed consent obtained from all patients.

**FIGURE 1:**
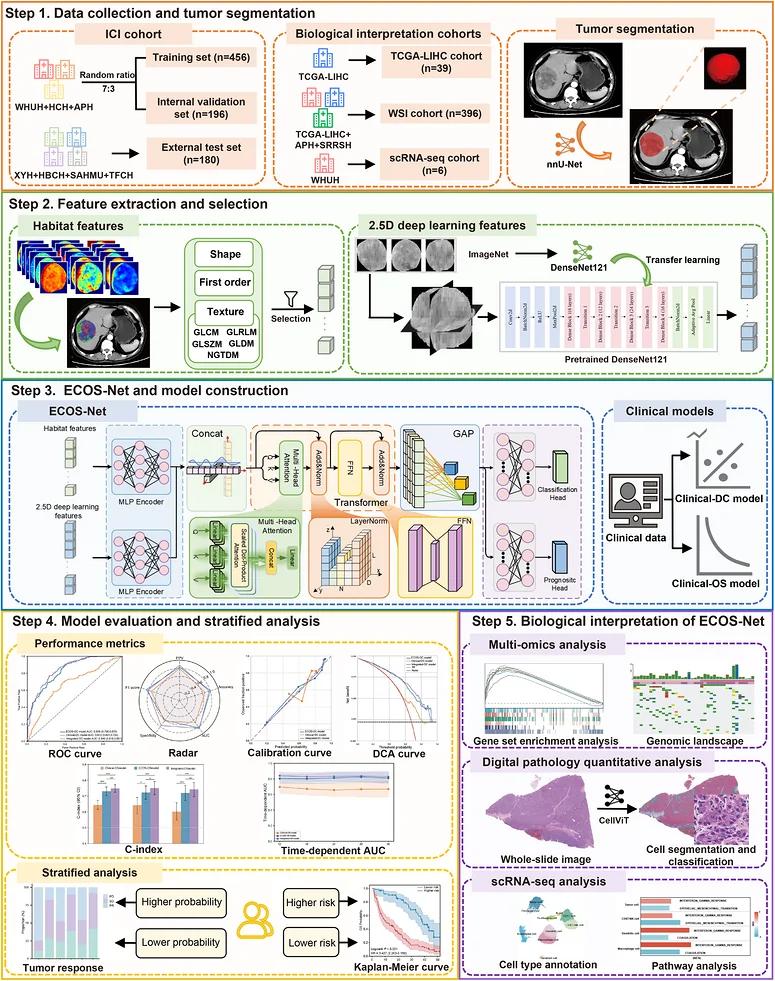
The workflow of the study.

Figure 1 illustrates the workflow of the study. We designed four cohorts to develop the multitask ECOS‐Net for concurrently predicting early DC and OS in HCC patients treated with ICIs and to explore potential biological correlates of ECOS‐Net predictions. Model development and validation were performed using the ICI cohort. Specifically, 652 patients from three hospitals were randomly divided in a 7:3 ratio into a training set (*n* = 456) and an internal validation set (*n* = 196), while the external test set comprised 180 patients from four additional hospitals. For exploratory biological interpretation, the TCGA‐LIHC cohort (*n* = 39) was used for multi‐omics analysis. The whole‐slide image (WSI) cohort (*n* = 396), including cases from TCGA‐LIHC and surgical resection cases from APH and SRRSH, was used for quantitative digital pathology. The scRNA‐seq cohort (*n* = 6) was used to characterize cellular composition and functional states. The details of the study design and cohorts are described in Appendix S1 and Figure S1.

### 2.2 Follow‐Up

Baseline and outcome data were obtained from electronic medical records and clinical follow‐up. Treatment regimens were recorded and categorized as ICIs alone, ICIs plus tyrosine kinase inhibitors (TKIs), or ICIs plus anti‐vascular endothelial growth factor (anti‐VEGF) agents according to the actual therapies administered. Tumor response and progression were determined based on clinical documentation and imaging assessments. Treatment response was evaluated using the first follow‐up CT performed 4–8 weeks after initiation of ICI therapy according to modified Response Evaluation Criteria in Solid Tumors (mRECIST) and categorized as complete response (CR), partial response (PR), stable disease (SD), or progressive disease (PD). Details of the mRECIST response assessment and central review are provided in Appendix S2. At the group level, efficacy was summarized as the objective response rate (ORR, proportion of patients with CR or PR) and the disease control rate (DCR, proportion with CR, PR, or SD). The primary endpoints were early DC and OS. Early DC was defined as the absence of PD (i.e., CR, PR, or SD) at the patient level. OS was defined as the time from initiation of ICI therapy to death from any cause, and progression‐free survival (PFS) as the time from treatment initiation to the first documented progression or death.

### 2.3 Image Preprocessing and Tumor Segmentation

Pretreatment contrast‐enhanced abdominal CT scans were retrieved from picture archiving and communication systems (PACS) and anonymized. Arterial‐phase images were standardized and resampled to isotropic voxels with a spatial resolution of 1 mm × 1 mm × 1 mm. Detailed CT acquisition parameters from the participating centers are provided in Table S1. Tumors were segmented using nnU‐Net [21] (https://github.com/MIC‐DKFZ/nnUNet) and reviewed by experienced abdominal radiologists, with manual correction when needed. Details are provided in Appendix S3.

### 2.4 Subregion Generation and Feature Extraction

Within the tumor volume of interest (VOI), voxel‐level features (Table S2) were clustered using K‐means to generate habitat subregions. The optimal cluster number (*k* = 3) was determined using the Calinski‐Harabasz and Davies‐Bouldin indices (Figure S2). Radiomic features were extracted using PyRadiomics (v3.0.1) and harmonized using ComBat (Figure S3). Feature selection was then performed to obtain 16 key habitat features (Figure S4). For 2.5D DL features, we adopted a tumor region‐of interest‐only strategy and constructed three‐channel 2.5D inputs (256 × 256) using orthogonal axial, sagittal, and coronal slices. An ImageNet‐pretrained DenseNet121 served as the backbone network. For each patient, 2.5D DL features were extracted from the global average pooling layer and subsequently reduced to 32 principal components using principal component analysis (PCA). Furthermore, gradient‐weighted class activation mapping (Grad‐CAM) was applied to DenseNet121 to provide visual interpretation of model decisions. Details of the subregion generation, feature extraction, and selection are provided in Appendix S4.

### 2.5 Model Development

**FIGURE 2:**
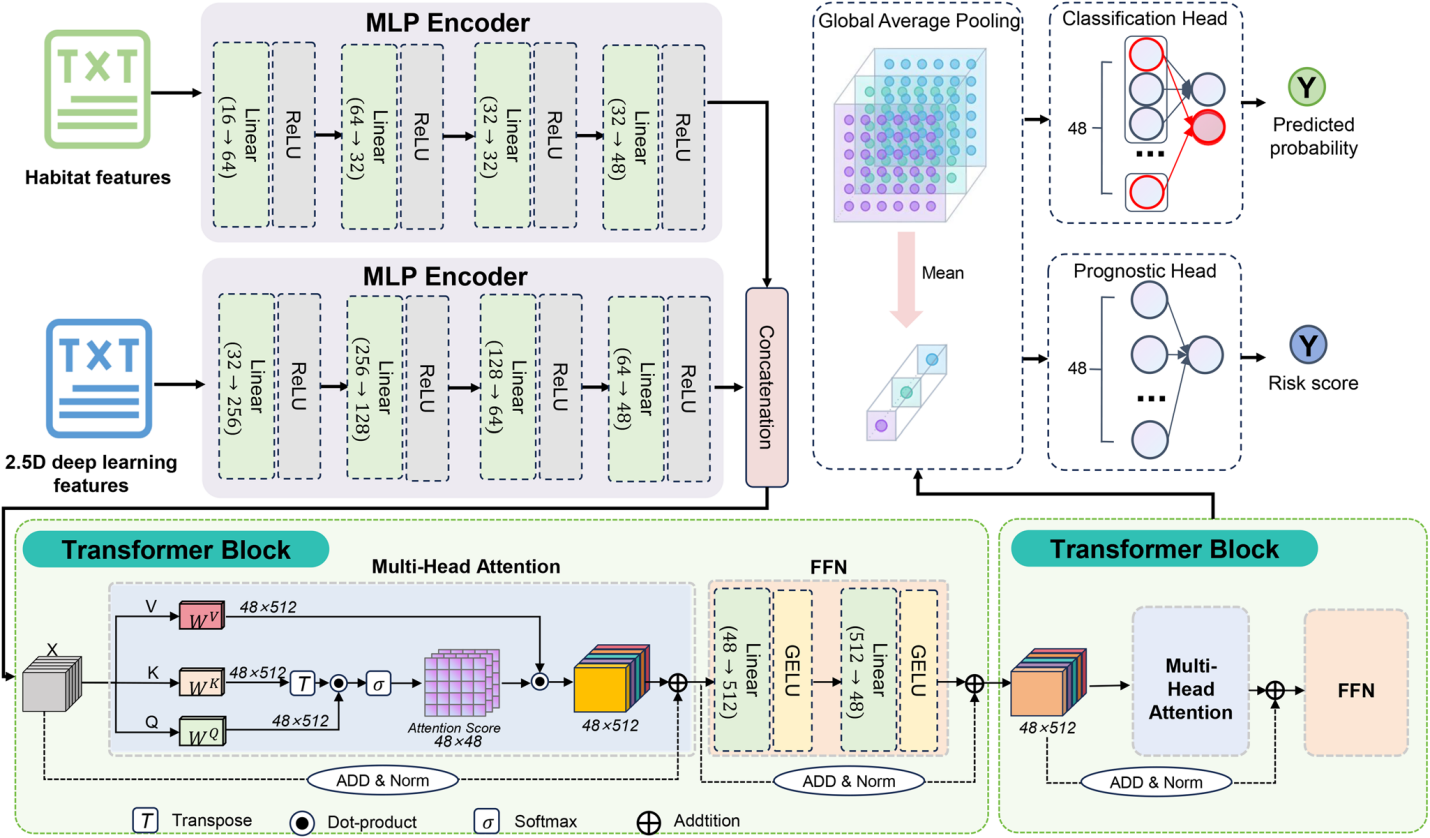
ECOS‐Net architecture diagram.

ECOS‐Net adopted a dual‐branch architecture that encoded pre‐extracted habitat and 2.5D DL features, respectively (Figure 2). Multi‐head attention was employed to achieve cross‐scale feature fusion. The task‐specific heads generated the predicted probability of early DC and the OS risk score, corresponding to the ECOS‐DC and ECOS‐OS models, respectively. In the training set, significant clinical variables were identified through regression analyses and used to construct the Clinical‐DC and Clinical‐OS models separately. Subsequently, predictions from the clinical models were integrated with the corresponding ECOS‐Net outputs to establish Integrated‐DC and Integrated‐OS models. The detailed methodology for model construction is provided in Appendix S5.

### 2.6 Model Evaluation

For early DC prediction, model discrimination was primarily assessed by the area under the receiver operating characteristic curve (AUC), with additional evaluation using specificity, accuracy, positive predictive value (PPV), and the F1 score. Calibration was assessed with calibration curves, and clinical utility was quantified across probability thresholds via decision curve analysis (DCA). For OS prediction, the prognostic performance of the models was evaluated using Harrell's concordance index (C‐index), supplemented by time‐dependent AUC analysis. Patients were stratified into higher and lower probability groups for early DC prediction using optimal thresholds determined in the training set. For OS prediction, they were similarly stratified into higher and lower risk groups. The independent associations of these stratifications with early DC or OS were evaluated using multivariable regression after adjustment for key clinical covariates, and their consistency was further assessed through subgroup analyses.

### 2.7 Biological Interpretation of ECOS‐Net

#### 2.7.1 Multi‐Omics Analysis

To explore the potential molecular correlates of ECOS‐Net predictions, we analyzed imaging‐matched multi‐omics data from the TCGA‐LIHC cohort. Patients were stratified into higher and lower probability groups (ECOS‐DC model) and higher and lower risk groups (ECOS‐OS model). Subsequently, we compared differential gene expression, pathway enrichment, immune infiltration, and other molecular characteristics between groups. The complete analytical methods are provided in Appendix S6.

#### 2.7.2 Digital Pathology Quantitative Analysis

An experienced pathologist first selected and annotated representative tumor slides in QuPath (https://qupath.github.io). Subsequently, our quantitative workflow involved nuclear segmentation and classification via the CellViT algorithm [22], followed by metric computation using a custom Groovy script (Table S3). We then explored potential pathological correlates of ECOS‐Net predictions. The full technical pipeline is detailed in Appendix S7.

#### 2.7.3 scRNA‐seq Analysis

This study used scRNA‐seq data from HCC surgical specimens to explore potential cellular features underlying ECOS‐Net predictions. The workflow comprised standard preprocessing, integration, clustering, and cell‐type annotation. Differential expression and pathway analyses were performed within each annotated cell type. Immune‐related functional programs (e.g., cytotoxicity, Treg activity, macrophage polarization) were quantified in key cell types using predefined gene signatures (Table S4) and compared between prediction groups. Comprehensive methodological parameters are detailed in Appendix S8.

### 2.8 Statistical Analysis

Continuous variables were presented as mean (standard deviation) or median (interquartile range), while categorical variables were expressed as frequency (percentage). Group comparisons were performed as follows: continuous variables were compared using the Student's *t*‐test or Wilcoxon rank‐sum test, and categorical variables using the χ^2^ test or Fisher's exact test. For early DC prediction, model discrimination was assessed by the AUC, with the DeLong test used for model comparison. The optimal classification threshold was determined in the training set via the Youden index and applied to the validation and test sets. Calibration and clinical utility were evaluated using calibration curves and DCA, respectively. For OS prediction, model performance was quantified using the C‐index, supplemented by time‐dependent AUC analysis. The optimal cutoff for risk stratification was identified in the training set using the “survminer” package and then fixed for application to the internal validation and external test sets to define higher and lower risk groups. OS and PFS were estimated by the Kaplan–Meier curves, and group differences were assessed using the log‐rank test. Multivariable logistic regression and Cox regression analyses were performed. Subgroup analyses were further performed to evaluate the consistency of ECOS‐Net performance. To address multiple comparisons from the evaluation of numerous biological parameters, *p* values were adjusted using the Benjamini‐Hochberg method where applicable, and adjusted *p* values (adj. *P* values) were reported. All tests were two‐sided, with *p* < 0.05 considered statistically significant. Statistical analyses were performed using R (version 4.5.0) and Python (version 3.9.7).

## 3 Results

### 3.1 Patient Characteristics

A total of 1234 HCC patients were ultimately included, comprising 832 patients who received ICI therapy and 402 who underwent surgery. The baseline demographic and clinical characteristics of the training, internal validation, and external test sets are presented in Table 1. Across these sets, most patients were male, had hepatitis B virus (HBV) infection, and were classified as Barcelona Clinic Liver Cancer (BCLC) stage C. Median OS was 13.10, 16.57, and 22.20 months in the training, internal validation, and external test sets, respectively. Median PFS was 5.57, 6.40, and 6.24 months in the corresponding sets. The ORRs were 24.1%, 31.6%, and 29.4%, and DCRs were 67.8%, 72.4%, and 71.1%, respectively (Table S5). Baseline information for the cohorts used for exploratory biological interpretation is summarized in Table S6. Central review of mRECIST‐based early response assessment demonstrated excellent inter‐reader agreement and high concordance with the original local assessment (Table S7).

**TABLE 1:** Baseline characteristics of patients in the training, internal validation, and external test sets.

Characteristics	Overall (*n* = 832)	Training set (*n* = 456)	Internal validation set (*n* = 196)	External test set (*n* = 180)
Age (years), n (%)				
< 60	472 (56.7%)	258 (56.6%)	111 (56.6%)	103 (57.2%)
≥ 60	360 (43.3%)	198 (43.4%)	85 (43.4%)	77 (42.8%)
Sex, n (%)				
Female	124 (14.9%)	75 (16.4%)	22 (11.2%)	27 (15%)
Male	708 (85.1%)	381 (83.6%)	174 (88.8%)	153 (85%)
BMI (kg/m^2^), median (IQR)	22.5 (20.5, 24.5)	22.6 (20.8, 24.7)	23.0 (20.7, 24.5)	21.3 (19.7, 24.0)
Types of ICIs, n (%)				
PD‐1	768 (92.3%)	423 (92.8%)	178 (90.8%)	167 (92.8%)
PD‐L1	56 (6.7%)	29 (6.4%)	15 (7.7%)	12 (6.7%)
PD‐1/CTLA‐4	8 (1%)	4 (0.9%)	3 (1.5%)	1 (0.6%)
Treatment				
ICIs alone	86 (10.3%)	59 (12.9%)	21 (10.7%)	6 (3.3%)
ICIs + TKIs	620 (74.5%)	321 (70.4%)	140 (71.4%)	159 (88.3%)
ICIs + anti‐VEGF	126 (15.1%)	76 (16.7%)	35 (17.9%)	15 (8.3%)
HBV infection, n (%)				
No	131 (15.7%)	56 (12.3%)	20 (10.2%)	55 (30.6%)
Yes	701 (84.3%)	400 (87.7%)	176 (89.8%)	125 (69.4%)
Cirrhosis, n (%)				
No	247 (29.7%)	125 (27.4%)	53 (27%)	69 (38.3%)
Yes	585 (70.3%)	331 (72.6%)	143 (73%)	111 (61.7%)
Tumor diameter (cm), median (IQR)	8 (4.8, 11.3)	8 (4.8, 11.2)	7.7 (5.0, 11.3)	8.6 (4.8, 12.1)
Tumor number, n (%)				
≤ 3	389 (46.8%)	208 (45.6%)	91 (46.4%)	90 (50%)
> 3	443 (53.2%)	248 (54.4%)	105 (53.6%)	90 (50%)
Macrovascular invasion, n (%)				
No	284 (34.1%)	155 (34%)	64 (32.7%)	65 (36.1%)
Yes	548 (65.9%)	301 (66%)	132 (67.3%)	115 (63.9%)
BCLC stage, n (%)				
B	168 (20.2%)	101 (22.1%)	40 (20.4%)	27 (15%)
C	664 (79.8%)	355 (77.9%)	156 (79.6%)	153 (85%)
Child‐Pugh, n (%)				
A	619 (74.4%)	348 (76.3%)	154 (78.6%)	117 (65%)
B	213 (25.6%)	108 (23.7%)	42 (21.4%)	63 (35%)
ALT (U/L), median (IQR)	39 (26, 61)	39 (27, 62)	42 (26, 60)	37.1 (24, 59)
AST (U/L), median (IQR)	51.5 (35, 76)	49.5 (34, 72)	48 (34, 76)	60.9 (39.8, 93.1)
PT (s), median (IQR)	13.7 (12.8, 14.9)	13.6 (12.8, 14.5)	13.6 (13, 14.5)	15.3 (12.4, 17.3)
NLR, median (IQR)	3.2 (2.1, 4.7)	3.3 (2.2, 4.8)	3.0 (2.1, 4.5)	2.8 (1.8, 4.3)
PLR, median (IQR)	126.5 (88.8, 176.9)	126.5 (88.2, 176.4)	120.8 (93.7, 174)	132.4 (88.2, 185.6)
ALBI, median (IQR)	−1.2 (−1.5, −0.94)	−1.2 (−1.4, −0.9)	−1.2 (−1.5, −1.0)	−1.3 (−1.5, −1.0)
AFP level (ng/mL), n (%)				
< 400	462 (55.5%)	267 (58.6%)	117 (59.7%)	78 (43.3%)
≥ 400	370 (44.5%)	189 (41.4%)	79 (40.3%)	102 (56.7%)

Abbreviations: AFP, alpha‐fetoprotein; ALBI, albumin‐bilirubin; ALT, alanine transaminase; anti‐VEGF, anti‐vascular endothelial growth factor; AST, aspartate transaminase; BCLC, Barcelona Clinic Liver Cancer; BMI, body mass index; HBV, hepatitis B virus; ICIs, immune checkpoint inhibitors;IQR, interquartile range; NLR, neutrophil‐to‐lymphocyte ratio; PLR, platelet‐to‐lymphocyte ratio; PT, prothrombin time; TKIs, tyrosine kinase inhibitors.

### 3.2 Feature Selection and Model Development

Sixteen habitat features were retained after feature selection. The 2.5D DL features were extracted from the global average pooling layer of DenseNet121 and reduced to 32 dimensions. In addition, Grad‐CAM maps were generated from DenseNet121 to provide visual interpretation of relevant image regions. Within ECOS‑Net, the habitat and 2.5D DL features were independently encoded by fully connected networks and projected into a unified 48‐dimensional embedding space. Feature fusion was performed via a transformer‐based multi‐head attention module. Finally, this pipeline enabled the simultaneous prediction of early DC and OS.

For the clinical models, multivariable logistic regression identified cirrhosis (OR = 0.61, 95% CI: 0.37–0.99, *p* = 0.047), increased tumor diameter (OR = 0.90, 95% CI: 0.85–0.95, *p* < 0.001), and tumor number > 3 (OR = 0.51, 95% CI: 0.34–0.78, *p* = 0.002) as independent factors associated with a lower probability of early DC, forming the basis for the Clinical‐DC model (Table S8). In multivariable Cox regression, age ≥ 60 years (HR = 1.37, 95% CI: 1.09–1.73, *p* = 0.008), increased tumor diameter (HR = 1.08, 95% CI: 1.05–1.11, *p* < 0.001), cirrhosis (HR = 1.32, 95% CI: 1.02–1.72, *p* = 0.038), and elevated ALT (HR = 1.00, 95% CI: 1.00–1.01, *p* = 0.036) were associated with shorter OS, while higher BMI was associated with longer OS (HR = 0.96, 95% CI: 0.93–0.99, *p* = 0.017) (Table S9), consistent with the reported “obesity paradox” [23]. To integrate imaging and clinical information, the predictions from ECOS‑Net were combined with those from the clinical models, yielding the Integrated‑DC and Integrated‑OS models.

### 3.3 Predictive Performance of the Models

**FIGURE 3:**
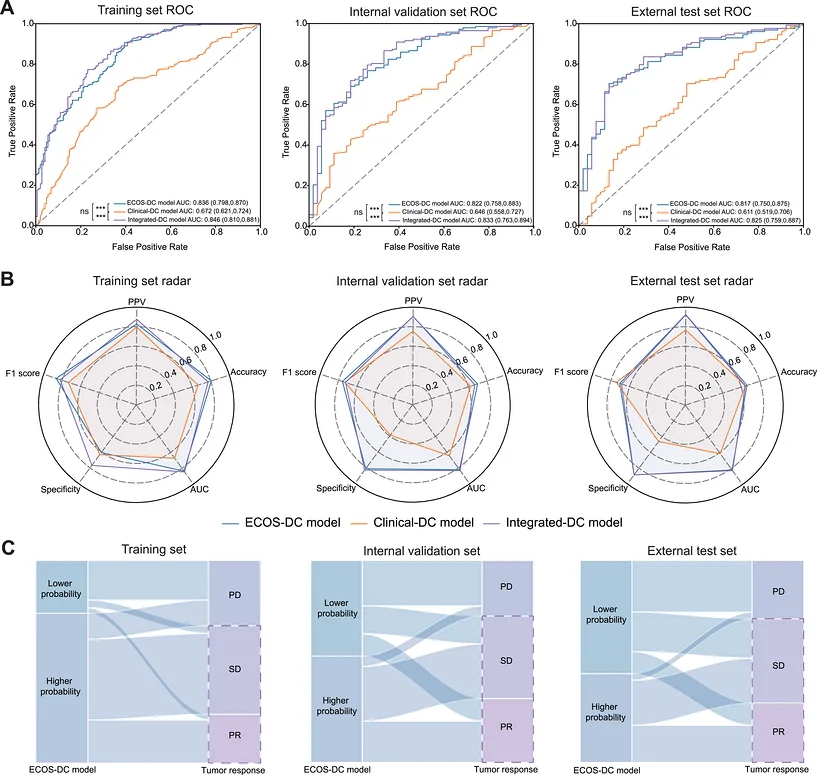
Model predictive performance and prediction distributions. (A) ROC curves of the Clinical‐DC, ECOS‐DC, and Integrated‐DC models in the training, internal validation, and external test sets. (B) Radar charts showing performance metrics across the three sets. (C) Sankey diagrams showing the flow distribution between different groups of the ECOS‐DC model and the actual tumor response. The dashed box indicates early disease control. ROC, receiver operating characteristic curve; PPV, positive predictive value; AUC, area under the receiver operating characteristic curve; PR, partial response; SD, stable disease; PD, progressive disease; ns, not significant; ^***^ *p* < 0.001.

The ECOS‐DC model achieved AUCs of 0.836 (95% CI: 0.798–0.870), 0.822 (95% CI: 0.758–0.883), and 0.817 (95% CI: 0.750–0.875) for predicting early DC in the training, internal validation, and external test sets, respectively (Figure 3A). The Integrated‐DC model yielded AUCs of 0.846 (95% CI: 0.810–0.881), 0.833 (95% CI: 0.763–0.894), and 0.825 (95% CI: 0.759–0.887) across the same sets. The Clinical‐DC model demonstrated lower AUCs of 0.672 (95% CI: 0.621–0.724), 0.646 (95% CI: 0.558–0.727), and 0.611 (95% CI: 0.519–0.706). Both the ECOS‐DC and Integrated‐DC models significantly outperformed the Clinical‐DC model in discriminative ability across all sets. Furthermore, the predicted probabilities from ECOS‑DC and Integrated‑DC were highly correlated (all *p* values < 0.001, Tables S10–S12). Other performance metrics are summarized in the radar charts (Figure 3B). The ECOS‑DC and Integrated‑DC models also showed good calibration (Figure S5A) and provided greater net clinical benefit than the clinical model across a wide threshold range in DCA analysis (Figure S5B). The Sankey diagrams illustrated the flow distribution between the predicted groups in the ECOS‐DC model and the actual tumor response status (Figure 3C).

**FIGURE 4:**
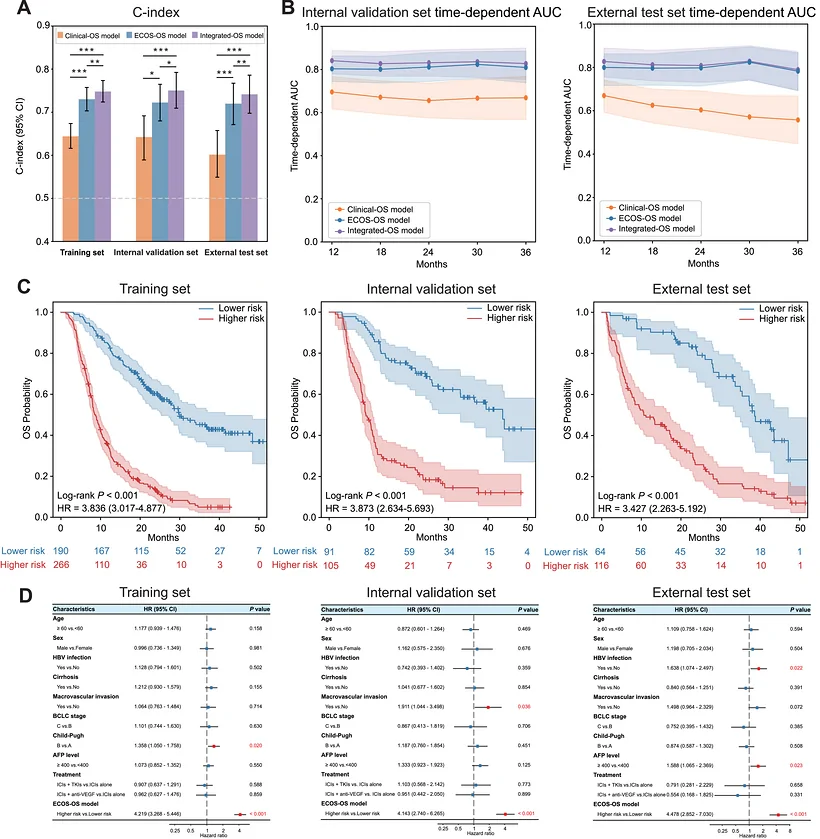
Model performance for overall survival prediction. (A) Bar charts of C‐indices for each model in the training, internal validation, and external test sets. (B) Time‐dependent AUCs in the internal validation and external test sets. (C) Kaplan‑Meier curves comparing OS between the higher risk and lower risk groups stratified by the ECOS‑OS model across all sets. (D) Multivariable Cox regression analyses of OS across all sets. C‐index, Harrell's concordance index; AUC, area under the receiver operating characteristic curve; OS, overall survival; HR, hazard ratio; CI, confidence interval; HBV, hepatitis B virus; AFP, alpha‐fetoprotein; ICIs, immune checkpoint inhibitors; TKIs, tyrosine kinase inhibitors; anti‐VEGF, anti‐vascular endothelial growth factor. ^*^ *p* < 0.05, ^**^ *p* < 0.01, ^***^ *p* < 0.001.

For OS prediction, the ECOS‐OS model attained C‐indices of 0.730 (95% CI: 0.703–0.757), 0.722 (95% CI: 0.680–0.765), and 0.720 (95% CI: 0.671–0.767) in the training, internal validation, and external test sets, respectively (Figure 4A). These C‐indices were consistently higher than those of the Clinical‐OS model, which yielded C‐indices of 0.644 (95% CI: 0.616–0.674), 0.642 (95% CI: 0.589–0.692), and 0.601 (95% CI: 0.549–0.657), respectively. The Integrated‐OS model achieved C‐indices of 0.748 (95% CI: 0.724–0.774), 0.750 (95% CI: 0.709–0.792), and 0.741 (95% CI: 0.698–0.786), respectively.

Time‐dependent AUCs in the internal validation and external test sets at 12, 18, 24, 30, and 36 months are shown in Figure 4B. The risk scores from ECOS‐OS and Integrated‐OS were positively correlated in all sets (all *p* values < 0.001, Tables S13–S15). Furthermore, ablation analyses showed that transformer‐based ECOS‐Net achieved numerically higher AUCs for early DC and C‐indices for OS than models using single‐scale imaging features or single‐task architectures (Tables S16 and S17).

### 3.4 Stratification and Subgroup Analyses

The distribution of clinical characteristics, survival data, and model predictions is summarized in Figure S6. When patients were stratified into higher and lower probability groups according to the ECOS‐DC model, tumor response distributions differed significantly between groups across all sets (Figure S7 and Table S18). ECOS‐DC and Integrated‐DC predicted probabilities were higher in the higher probability groups than in the lower probability groups (Figure S8). After adjusting for clinical variables, the ECOS‐DC‐defined higher probability group was still independently associated with a higher likelihood of early DC in both the internal validation and external test sets (Table S19). Subgroup analyses generally supported directionally consistent associations of ECOS‐DC stratification across various subgroups in the internal validation and external test sets (Figure S9), although the PD‐1/CTLA‐4 therapy group was not analyzed separately because of limited sample size. Statistical testing was not feasible in a few subgroups due to the absence of outcome variation in the higher probability group, where all cases achieved early DC. The *p* values for interaction between ECOS‐DC stratification and treatment regimen were 0.077 and 0.748 in the internal validation and external test sets, respectively. Performance metrics (AUC, accuracy, specificity, F1 score, PPV) for each subgroup are detailed in Table S20.

Kaplan‐Meier curves showed that the higher risk groups defined by ECOS‐OS (Figure 4C), Clinical‐OS, and Integrated‐OS exhibited significantly shorter OS and PFS than their corresponding lower risk groups, except that PFS stratification by the Clinical‐OS model did not reach statistical significance in the internal validation set (Figure S10). The calibration curves and decision curve analyses of the survival models are shown for the 1‐, 2‐, and 3‐year predictions in Figures S11 and S12, respectively. Multivariable Cox regression showed that the ECOS‐OS higher risk group remained independently associated with shorter OS (Figure 4D) and PFS (Table S21). Subgroup analyses of the ECOS‐OS model indicated that, in both the internal validation and external test sets, the higher risk groups were directionally associated with worse OS and PFS across most subgroups (Figures S13 and S14). The *p* values for interaction between ECOS‐OS risk stratification and treatment regimen were 0.355 and 0.577 for OS and 0.405 and 0.895 for PFS in the internal validation and external test sets, respectively. Similar subgroup analyses for the Integrated‑OS model are presented in Figures S15 and S16, with *p* values for interaction between model‐defined risk stratification and treatment regimen of 0.130 and 0.513 for OS and 0.702 and 0.975 for PFS in the internal validation and external test sets, respectively. Exploratory stratified sensitivity analyses by center and CT acquisition parameters showed generally similar AUCs for ECOS‐DC and C‐indices for ECOS‐OS across evaluable subgroups, although several subgroups had small sample sizes or no evaluable cases (Table S22).

### 3.5 Biological Interpretation of ECOS‐Net

#### 3.5.1 Multi‐Omics Analysis

**FIGURE 5:**
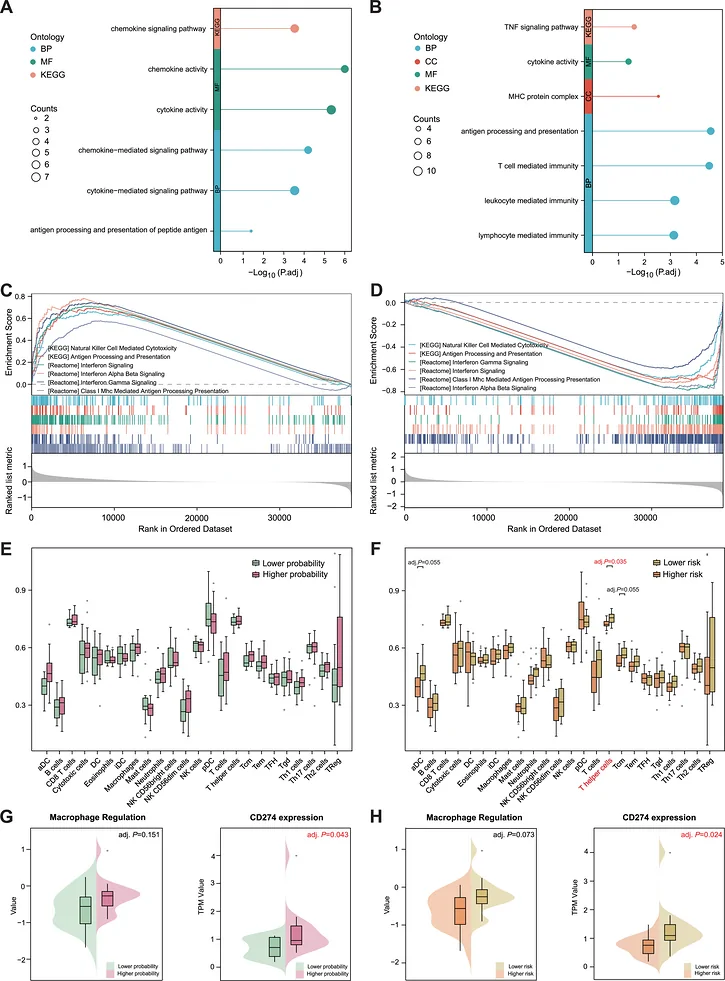
Exploratory biological characterization of the ECOS‐DC and ECOS‐OS prediction groups. (A) GO and KEGG enrichment analyses of nominal DEGs upregulated in the higher probability group compared with the lower probability group of the ECOS‐DC model. (B) GO and KEGG enrichment analyses of nominal DEGs downregulated in the higher risk group compared with the lower risk group of the ECOS‐OS model. (C) GSEA analyses of enriched pathways in the higher probability group compared with the lower probability group in the ECOS‐DC model. (D) GSEA analyses of negatively enriched pathways in the higher risk group compared with the lower risk group in the ECOS‐OS model. (E) Comparison of immune cell signature enrichment between lower and higher probability groups of the ECOS‐DC model. (F) Comparison of immune cell signature enrichment between lower and higher risk groups of the ECOS‐OS model. (G) Comparison of macrophage regulation score and CD274 (PD‐L1) expression between lower and higher probability groups of the ECOS‐DC model. (H) Comparison of macrophage regulation score and CD274 (PD‐L1) expression between lower and higher risk groups of the ECOS‐OS model. Pathway‐level significance and biological comparisons were assessed after Benjamini‐Hochberg adjustment. DEGs, differentially expressed genes; BP, biological process; CC, cellular component; MF, molecular function.

Differential expression analysis identified 51 nominally defined differentially expressed genes (DEGs) (39 upregulated and 12 downregulated) between the ECOS‐DC higher and lower probability groups, and 128 nominal DEGs (48 upregulated and 80 downregulated) between the ECOS‐OS higher and lower risk groups. Gene Ontology (GO) and Kyoto Encyclopedia of Genes and Genomes (KEGG) analyses showed that nominal DEGs upregulated in the higher probability group and those downregulated in the higher risk group were enriched in inflammatory and immune‐related pathways (Figure 5A,B), whereas the opposite gene sets (downregulated in the higher probability and upregulated in the higher risk groups) were enriched in pathways related to cholesterol and lipoprotein metabolism (Figure S17). Gene set enrichment analysis (GSEA) using KEGG and Reactome terms further supported this pattern, showing enrichment of immune‐related pathways in the higher probability group and reduced enrichment of these pathways in the higher risk group (Figure 5C,D). Pathway‐level significance in both GO/KEGG and GSEA analyses was assessed after Benjamini‐Hochberg adjustment.

The immune cell signature enrichment in different groups of the ECOS‐DC model is shown in Figure 5E. In the ECOS‐OS model, T helper cell signature scores were significantly higher in the lower risk group (adj. *P* = 0.035), while activated dendritic cell (aDC) and central memory T cell (Tcm) signature scores exhibited borderline trends toward higher levels in the lower risk group (Figure 5F). MCP‐counter analysis yielded a similar pattern for natural killer (NK) cell abundance, with higher values in the ECOS‐DC higher probability group and the ECOS‐OS lower risk group, although neither comparison reached statistical significance after correction (Figure S18). Macrophage regulation and other immune scores were not significantly different between groups (Tables S23 and S24). At the biomarker level, CD274 (PD‑L1) expression was elevated in the ECOS‐DC higher probability group (adj. *P* = 0.043, Figure 5G) and the ECOS‐OS lower risk group (adj. *P* = 0.024, Figure 5H), whereas TMB and MSI MANTIS scores did not differ significantly between groups. Figure S19 also presents expression of common immune checkpoint genes and the top 30 mutated genes in the TCGA‐LIHC cohort.

#### 3.5.2 Digital Pathology Quantitative Analysis

**FIGURE 6:**
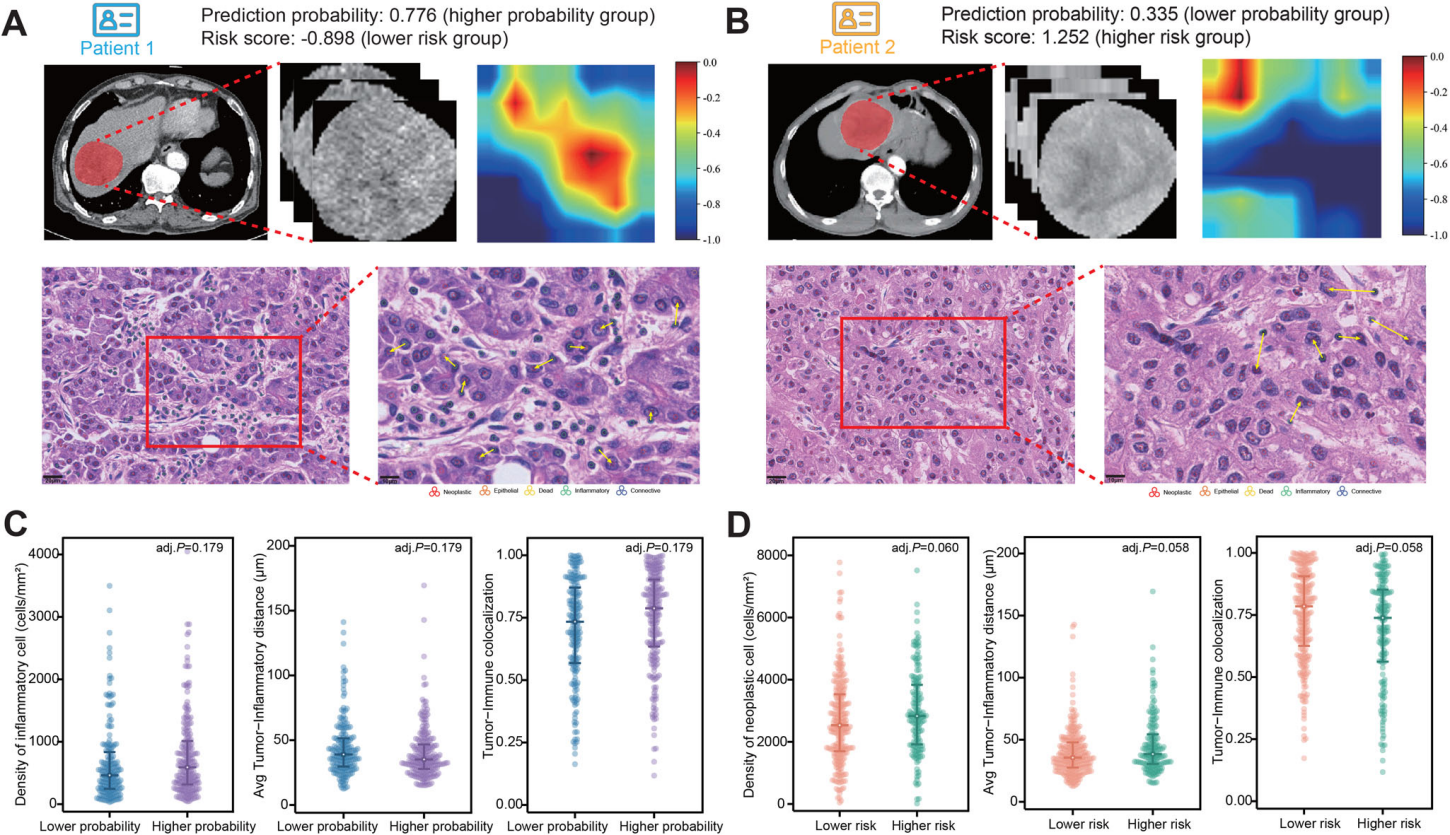
Representative HCC patients and WSI analysis stratified by ECOS‐DC and ECOS‐OS models. (A) An HCC patient with an ECOS‐DC predicted probability of 0.776 and an ECOS‐OS predicted risk score of ‐0.898 was classified into the higher probability and lower risk groups. (B) An HCC patient with an ECOS‐DC predicted probability of 0.335 and an ECOS‐OS predicted risk score of 1.252 was classified into the lower probability and higher risk groups. Grad‐CAM was applied to DenseNet121 to provide a visual interpretation of its predictions. The yellow arrow indicates the distance between neoplastic cells and inflammatory cells. (C) Comparison of pathological quantitative metrics between groups stratified by the ECOS‐DC model. (D) Comparison of pathological quantitative metrics between groups stratified by the ECOS‐OS model.

Representative pathological findings from two patients with HCC are presented in Figure 6A,B. WSI‐derived quantitative parameters showed nominal trends toward higher inflammatory cell density, greater tumor‐immune colocalization, and shorter tumor‐inflammatory distance in the ECOS‐DC higher probability group, although none of these parameters reached statistical significance after adjustment (Figure 6C and Table S25). In the ECOS‐OS model, the higher risk group tended to exhibit higher neoplastic cell density, greater tumor‐inflammatory distance, and lower tumor‐immune colocalization than the lower risk group, with borderline adjusted *P* values (Figure 6D and Table S26).

#### 3.5.3 scRNA‐seq Analysis

In six scRNA‐seq HCC samples, the stratification results of the ECOS‐DC and ECOS‐OS models were highly consistent: the ECOS‐DC higher probability group corresponded to the ECOS‐OS lower risk group, whereas the ECOS‐DC lower probability group corresponded to the ECOS‐OS higher risk group. Therefore, subsequent scRNA‐seq analyses were performed using this unified stratification (higher probability‐lower risk vs. lower probability‐higher risk).

**FIGURE 7:**
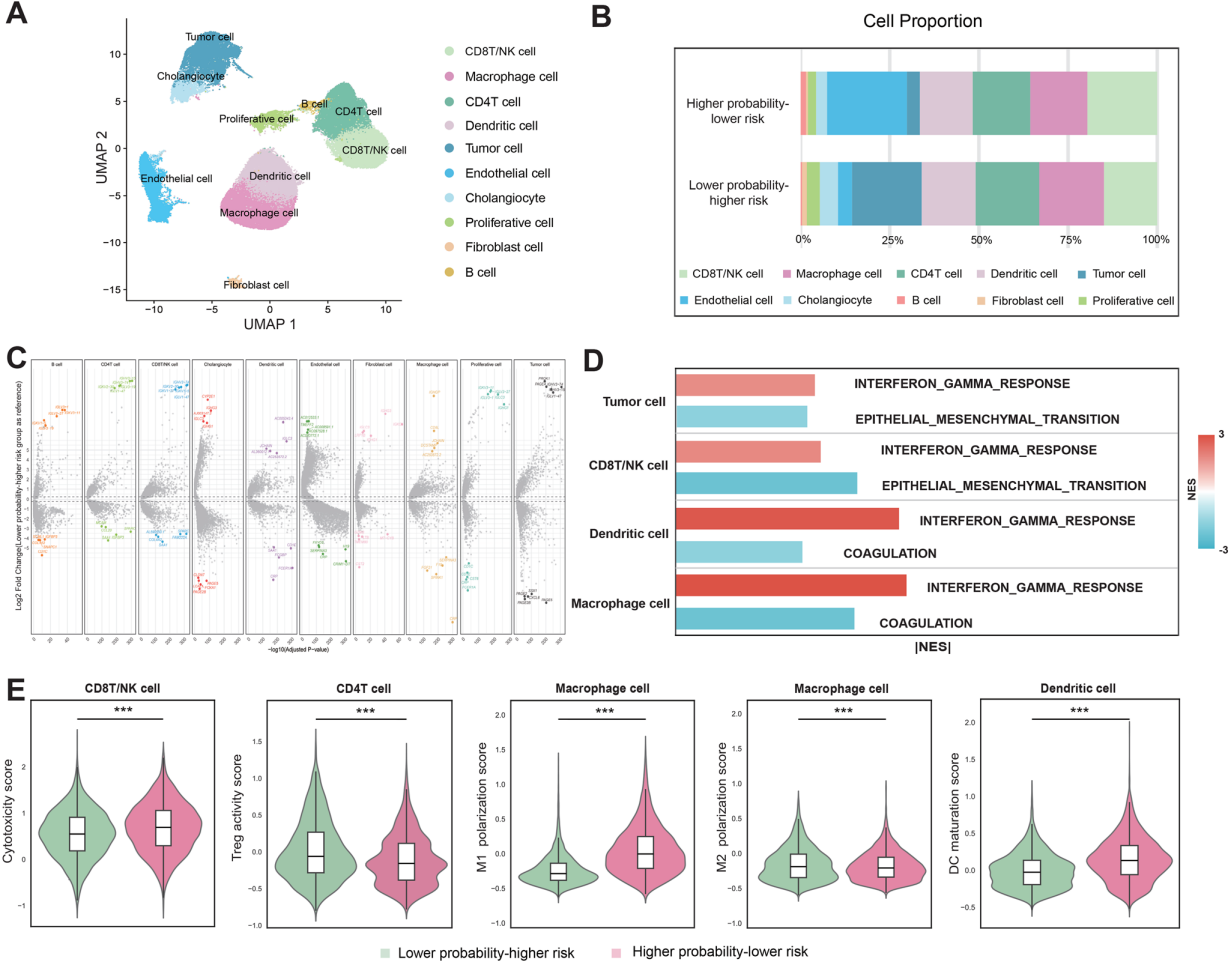
scRNA‐seq analysis of the tumor immune microenvironment. (A) UMAP visualization of 10 major cell types. (B) The proportions of cell types between higher probability‐lower risk and lower probability‐higher risk groups. (C) Volcano plots of differentially expressed genes across cell types. (D) GSEA analyses of biological pathways in the higher probability‐lower risk group compared with the lower probability‐higher risk group in the cell types. (E) Comparison of immune‐related signature scores in specific cell types between the two stratified groups. NES, normalized enrichment score; ^***^adj. *P* < 0.001.

Cells were annotated into 10 major cell populations based on classical marker genes (Figure S20), with the annotation results and group distributions shown in Figure 7A,B. Figure 7C also displays the DEGs for each cell type. In these exploratory analyses, compared with the lower probability‐higher risk group, the higher probability‐lower risk group showed enrichment of interferon‑related pathways across multiple cell types, including tumor cells, CD8^+^ T/NK cells, dendritic cells, and macrophages (Figure 7D and Figure S21). In addition, the higher probability‐lower risk group was also associated with higher cytotoxicity in CD8^+^ T/NK cells, lower Treg activity scores in CD4^+^ T cells, higher M1 and lower M2 polarization scores in macrophages, and increased dendritic cell maturation (Figure 7E).

## 4 Discussion

Accurate prediction of early DC and OS is crucial for optimizing therapeutic strategies and refining prognostic evaluation in patients with HCC receiving immunotherapy. In this study, we developed ECOS‐Net, a transformer‐based multitask framework that integrated CT‐derived habitat features and 2.5D DL features to simultaneously predict early DC and OS. ECOS‐DC achieved AUCs of 0.817–0.836, whereas ECOS‐OS yielded C‐indices of 0.720–0.730 across the training, internal validation, and external test sets, outperforming the corresponding clinical models. In addition, ECOS‐Net‐derived stratification remained independently associated with clinical outcomes after multivariable adjustment. Exploratory biological analyses suggested potential immune‐related correlates of ECOS‐Net predictions. These findings suggested that ECOS‐Net is a promising non‐invasive risk stratification framework for concurrent assessment of early treatment response and long‐term prognosis in HCC immunotherapy, although prospective validation remains necessary before clinical implementation.

ITH profoundly influences tumor progression, therapeutic resistance, and recurrence [4, 5]. It also serves as a key biological driver of the variable response to immunotherapy in HCC. Radiomics has emerged as a non‑invasive tool to characterize ITH and predict clinical outcomes [24]. However, conventional radiomic models primarily relied on whole‐tumor features, which insufficiently reflected the complex spatial heterogeneity within tumors [25]. In this study, we employed a complementary multi‐scale imaging strategy to characterize ITH by using habitat analysis to delineate intratumoral subregions and extract spatial heterogeneity features, while using 2.5D DL to learn high‐level imaging features from multiplanar CT information. A transformer‐based multi‐head attention mechanism was then adopted to integrate features across scales. The results showed that the ECOS‐DC and ECOS‐OS models derived from ECOS‐Net not only significantly outperformed the clinical models but also achieved higher performance than single‐scale imaging models and single‐task models. These findings supported the value of integrating complementary ITH‐related imaging information within a multitask framework for outcome prediction in HCC immunotherapy.

Early DC reflects the initial tumor response to immunotherapy, where stable disease represents a meaningful clinical state that identifies potential beneficiaries and preserves a therapeutic window for subsequent interventions. In contrast, OS remains the gold‐standard endpoint for long‐term benefit. Therefore, concurrent prediction of early DC and OS may provide a more comprehensive assessment of treatment benefit and support individualized risk stratification. In this study, ECOS‐Net demonstrated favorable dual‐endpoint predictive performance across the training, internal validation, and external test sets, with ECOS‐DC achieving AUCs of 0.836, 0.822, and 0.817 and ECOS‐OS yielding C‐indices of 0.730, 0.722, and 0.720, respectively. After adjusting for clinical variables, ECOS‐Net‐derived stratification remained independently associated with both early DC and OS, suggesting that ECOS‐Net provided independent predictive value beyond conventional clinical factors. Treatment heterogeneity is also an important consideration in real‐world HCC immunotherapy [26]. Different combination regimens could influence early tumor response and survival through effects on tumor angiogenesis, vascular normalization, immune‐cell infiltration, and disease progression [27, 28]. Subgroup analyses in both the internal validation and external test sets suggested directionally consistent associations across most clinical and treatment subgroups. The interaction analyses did not provide strong evidence of treatment‐related heterogeneity. These findings supported the potential role of ECOS‐Net as an imaging‐based risk stratification framework for HCC immunotherapy, while its clinical applicability requires further validation in prospective studies before routine implementation.

The TCGA‐LIHC, WSI, and scRNA‐seq cohorts were used to explore potential biological correlates of ECOS‐Net predictions. In the TCGA‐LIHC cohort, the ECOS‐DC higher probability group and the ECOS‐OS lower risk group were associated with enrichment of inflammatory and immune‐related pathways, as well as elevated CD274 (PD‐L1) expression, an observation broadly consistent with previous reports describing immune‐inflamed tumor phenotypes and an immune‐specific molecular class of HCC with potential relevance to immunotherapy responsiveness [29, 30]. The higher T helper cell signature score in the ECOS‐OS lower risk group may reflect T‐cell supportive immune features [31], with possible relevance to OS. Although several immune and pathological features did not remain significant after correction, WSI‐derived analyses showed trends toward shorter tumor‐inflammatory distance and greater tumor‐immune colocalization in the lower ECOS‐OS risk group. These findings might suggest closer spatial contact between inflammatory and tumor cells [32], potentially providing supportive pathological context for ECOS‐Net predictions. At the cellular level, the higher probability‐lower risk group showed a pattern suggestive of enhanced immune‐effector activity and reduced immunosuppression. Overall, these exploratory findings suggested that ECOS‐Net predictions were associated with immune‐related tumor microenvironmental features.

This study has several limitations. First, although this study included data from multiple centers, its retrospective nature may have introduced selection and information biases, and minor segmentation variability and residual CT acquisition heterogeneity could not be fully excluded. The exploratory CT acquisition sensitivity analyses included some subgroups with small sample sizes or no evaluable cases, while several treatment regimen subgroups also had relatively few patients or limited outcome variation. Therefore, larger prospective multicenter cohorts with standardized imaging protocols and segmentation workflows, and adequate sample sizes and sufficient outcome variation across CT acquisition and treatment regimen subgroups, are needed to confirm the generalizability of ECOS‐Net. Second, early DC was assessed at the first imaging evaluation 4–8 weeks after ICI initiation, and atypical response patterns such as delayed response or pseudoprogression may not have been fully captured. Prospective studies should incorporate multi‐time‐point imaging to better characterize post‐ICI response trajectories. In addition, this study used only arterial‐phase CT and selected the largest measurable lesion for multifocal tumors. Future work should explore multiphasic CT and multi‐lesion feature fusion strategies to more comprehensively capture ITH‐related imaging information. Finally, the biological interpretation cohorts were not derived from ICI‐treated patients. Therefore, further validation in larger ICI‐treated cohorts with matched molecular and cellular data is needed.

## 5 Conclusion

This study developed ECOS‐Net, a transformer‐based multitask DL framework integrating CT‐derived habitat and 2.5D DL features for simultaneous prediction of early DC and OS in HCC patients receiving immunotherapy. ECOS‐Net showed favorable performance across multicenter training, internal validation, and external test sets. Exploratory biological analyses suggested that ECOS‐Net predictions were associated with immune‐related tumor microenvironment features. Therefore, ECOS‐Net holds promise as a non‐invasive imaging‐based risk stratification framework for concurrently predicting early DC and OS in HCC patients treated with ICIs, with future prospective validation needed to support its clinical and biological implications.

## Author Contributions

X.N.F., B.X.G. and J.L. were involved in conceptualization, data curation, formal analysis and writing – original draft. Y.S.G. was involved in conceptualization, funding acquisition, formal analysis and writing – original draft. N.W., X.J.C., H.T.H., R.D.W., X.F.G., S.M.L., Y.Y.X., S.C.L., M.S.L., S.B.P., W.S., Z.G.Z. and Z.F.S. participated in data curation and investigation. P.C.L., X.Y.L. and P.S. were responsible for software, methodology and investigation. G.L.Z. and S.S.J. participated in data curation and visualization. J.X.Z. contributed to project administration, supervision, and writing – review & editing. L.Y. contributed to funding acquisition, project administration, and supervision.

## Conflicts of Interest

The authors declare no conflicts of interest.

## Supporting information




**Supporting File**: advs78005‐sup‐0001‐SuppMat.docx.

## Data Availability

Data will be available upon reasonable request to the corresponding author (Lian Yang).

## References

[1] F. Bray , M. Laversanne , H. Sung , et al., “Global Cancer Statistics 2022: GLOBOCAN Estimates of Incidence and Mortality Worldwide for 36 Cancers in 185 Countries,” CA: A Cancer Journal for Clinicians 74, no. 3 (2024): 229–263, 10.3322/caac.21834. 38572751

[2] M. Pinter , B. Scheiner , and D. J. Pinato , “Immune Checkpoint Inhibitors in Hepatocellular Carcinoma: Emerging Challenges in Clinical Practice,” The Lancet Gastroenterology & Hepatology 8, no. 8 (2023): 760–770, 10.1016/s2468-1253(23)00147-4. 37327807

[3] J. M. Llovet , F. Castet , M. Heikenwalder , et al., “Immunotherapies for Hepatocellular Carcinoma,” Nature Reviews Clinical Oncology 19, no. 3 (2022): 151–172, 10.1038/s41571-021-00573-2. 34764464

[4] A. Marusyk , M. Janiszewska , and K. Polyak , “Intratumor Heterogeneity: The Rosetta Stone of Therapy Resistance,” Cancer Cell 37, no. 4 (2020): 471–484, 10.1016/j.ccell.2020.03.007. PMC7181408 32289271

[5] N. McGranahan and C. Swanton , “Clonal Heterogeneity and Tumor Evolution: Past, Present, and the Future,” Cell 168, no. 4 (2017): 613–628, 10.1016/j.cell.2017.01.018. 28187284

[6] K. Chen , T. W. H. Shuen , and P. K. H. Chow , “The Association Between Tumour Heterogeneity and Immune Evasion Mechanisms in Hepatocellular Carcinoma and Its Clinical Implications,” British Journal of Cancer 131, no. 3 (2024): 420–429, 10.1038/s41416-024-02684-w. PMC11300599 38760445

[7] B. Losic , A. J. Craig , C. Villacorta‐Martin , et al., “Intratumoral Heterogeneity and Clonal Evolution in Liver Cancer,” Nature Communications 11, no. 1 (2020): 291, 10.1038/s41467-019-14050-z. PMC6962317 31941899

[8] Z.‐F. Lin , L.‐X. Qin , and J.‐H. Chen , “Biomarkers for Response to Immunotherapy in Hepatobiliary Malignancies,” Hepatobiliary & Pancreatic Diseases International 21, no. 5 (2022): 413–419, 10.1016/j.hbpd.2022.08.002. 35973935

[9] D. T. Le , J. N. Durham , K. N. Smith , et al., “Mismatch Repair Deficiency Predicts Response of Solid Tumors to PD‐1 Blockade,” Science 357, no. 6349 (2017): 409–413, 10.1126/science.aan6733. PMC5576142 28596308

[10] M. Vithayathil , D. Koku , C. Campani , et al., “Machine Learning Based Radiomic Models Outperform Clinical Biomarkers in Predicting Outcomes After Immunotherapy for Hepatocellular Carcinoma,” Journal of Hepatology 83, no. 4 (2025): 959–970, 10.1016/j.jhep.2025.04.017. 40246150

[11] Y. Hua , Z. Sun , Y. Xiao , et al., “Pretreatment CT‐based Machine Learning Radiomics Model Predicts Response in Unresectable Hepatocellular Carcinoma Treated With Lenvatinib plus PD‐1 Inhibitors and Interventional Therapy,” Journal for ImmunoTherapy of Cancer 12, no. 7 (2024): 008953, 10.1136/jitc-2024-008953. PMC11261678 39029924

[12] S. M. Kalasekar , C. H. VanSant‐Webb , and K. J. Evason , “Intratumor Heterogeneity in Hepatocellular Carcinoma: Challenges and Opportunities,” Cancers 13, no. 21 (2021): 5524, 10.3390/cancers13215524. PMC8582820 34771685

[13] Y. Zhang , S. Wang , M. Song , et al., “MRI‐based Intra‐ and Peritumoral Heterogeneity in Hepatocellular Carcinoma for Microvascular Invasion Prediction and Prognostic Risk Stratification,” Radiology: Imaging Cancer 7 (2025): 250066, 10.1148/rycan.250066. PMC12670032 41134139

[14] X. Shen , J.‐X. Zhang , H.‐T. Yan , et al., “CT‐Based Habitat Model for Predicting Tumor Response and Survival in Hepatocellular Carcinoma Treated With Transarterial Chemoembolization Combining Molecular Targeted Agents and Immune Checkpoint Inhibitors,” Academic Radiology 32, no. 12 (2025): 7095–7107, 10.1016/j.acra.2025.09.022. 41058349

[15] K. Chen , C. Sui , Z. Wang , Z. Liu , L. Qi , and X. Li , “Habitat Radiomics Based on CT Images to Predict Survival and Immune Status in Hepatocellular Carcinoma, a Multi‐cohort Validation Study,” Translational Oncology 52 (2025): 102260, 10.1016/j.tranon.2024.102260. PMC11754828 39752907

[16] Y. Xia , J. Zhou , X. Xun , et al., “CT‐Based Multimodal Deep Learning for Non‐Invasive Overall Survival Prediction in Advanced Hepatocellular Carcinoma Patients Treated With Immunotherapy,” Insights into Imaging 15, no. 1 (2024): 214, 10.1186/s13244-024-01784-8. PMC11347550 39186192

[17] Z. Lin , W. Wang , Y. Yan , Z. Ma , Z. Xiao , and K. Mao , “A Deep Learning‐Based Clinical‐radiomics Model Predicting the Treatment Response of Immune Checkpoint Inhibitors (ICIs)‐Based Conversion Therapy in Potentially Convertible Hepatocelluar Carcinoma Patients: A Tumor Marker Prognostic Study,” International Journal of Surgery 111, no. 5 (2025): 3342–3355, 10.1097/js9.0000000000002322. PMC12165573 40085751

[18] A. Vaswani , N. Shazeer , N. Parmar , et al., “Attention Is All You Need,” Advances in Neural Information Processing Systems 30 (2017): 5998–6008.

[19] J. Chen , J. Chen , Y. Ye , et al., “Pretreatment Multi‐sequence Contrast‐Enhanced MRI to Predict Response to Immunotherapy in Unresectable Hepatocellular Carcinoma Using Transformer: A Multicenter Study,” Journal of Cancer 16, no. 8 (2025): 2663–2672, 10.7150/jca.111026. PMC12170994 40535816

[20] K. He , C. Gan , Z. Li , et al., “Transformers in Medical Image Analysis,” Intelligent Medicine 3, no. 1 (2023): 59–78.

[21] F. Isensee , P. F. Jaeger , S. A. A. Kohl , J. Petersen , and K. H. Maier‐Hein , “nnU‐Net: A Self‐Configuring Method for Deep Learning‐Based Biomedical Image Segmentation,” Nature Methods 18, no. 2 (2021): 203–211, 10.1038/s41592-020-01008-z. 33288961

[22] F. Hörst , M. Rempe , L. Heine , et al., “CellViT: Vision Transformers for Precise Cell Segmentation and Classification,” Medical Image Analysis 94 (2024): 103143, 10.1016/j.media.2024.103143. 38507894

[23] M. Gallo , V. Adinolfi , V. Barucca , et al., “Expected and Paradoxical Effects of Obesity on Cancer Treatment Response,” Reviews in Endocrine and Metabolic Disorders 22, no. 4 (2021): 681–702, 10.1007/s11154-020-09597-y. 33025385

[24] M. Á. Berbís , F. P. Godino , J. Rodríguez‐Comas , et al., “Radiomics in CT and MR Imaging of the Liver and Pancreas: Tools With Potential for Clinical Application,” Abdominal Radiology 49 (2024): 322–340, 10.1007/s00261-023-04071-0. 37889265

[25] Z. Feng , H. Li , Q. Liu , et al., “CT Radiomics to Predict Macrotrabecular‐Massive Subtype and Immune Status in Hepatocellular Carcinoma,” Radiology 307, no. 1 (2023): e221291, 10.1148/radiol.221291. 36511807

[26] L. Rimassa , R. S. Finn , and B. Sangro , “Combination Immunotherapy for Hepatocellular Carcinoma,” Journal of Hepatology 79, no. 2 (2023): 506–515, 10.1016/j.jhep.2023.03.003. 36933770

[27] D. Fukumura , J. Kloepper , Z. Amoozgar , D. G. Duda , and R. K. Jain , “Enhancing Cancer Immunotherapy Using Antiangiogenics: Opportunities and Challenges,” Nature Reviews Clinical Oncology 15, no. 5 (2018): 325–340, 10.1038/nrclinonc.2018.29. PMC5921900 29508855

[28] W. S. Lee , H. Yang , H. J. Chon , and C. Kim , “Combination of Anti‐angiogenic Therapy and Immune Checkpoint Blockade Normalizes Vascular‐immune Crosstalk to Potentiate Cancer Immunity,” Experimental & Molecular Medicine 52, no. 9 (2020): 1475–1485, 10.1038/s12276-020-00500-y. PMC8080646 32913278

[29] V. Thorsson , D. L. Gibbs , S. D. Brown , et al., “The Immune Landscape of Cancer,” Immunity 48, no. 4 (2018): 812–830.e14, 10.1016/j.immuni.2018.03.023. PMC5982584 29628290

[30] D. Sia , Y. Jiao , I. Martinez‐Quetglas , et al., “Identification of an Immune‐Specific Class of Hepatocellular Carcinoma, Based on Molecular Features,” Gastroenterology 153, no. 3 (2017): 812–826, 10.1053/j.gastro.2017.06.007. PMC12166766 28624577

[31] J. Borst , T. Ahrends , N. Bąbała , C. J. M. Melief , and W. Kastenmüller , “CD^4+^ T cell help in cancer immunology and immunotherapy,” Nature Reviews Immunology 18, no. 10 (2018): 635–647, 10.1038/s41577-018-0044-0. 30057419

[32] E. Maestri , N. Kedei , S. Khatib , et al., “Spatial Proximity of Tumor‐Immune Interactions Predicts Patient Outcome in Hepatocellular Carcinoma,” Hepatology 79, no. 4 (2024): 768–779, 10.1097/hep.0000000000000600. PMC10948323 37725716

