# Supplementary material for: Transformer‐Based Multitask Framework Integrating Habitat and Deep Learning for Predicting Early Disease Control and Survival in Immunotherapy‐Treated Hepatocellular Carcinoma
Source: Adv Sci (Weinh). 2026 Sep 27:e78005. Online ahead of print. doi: 10.1002/advs.78005 (PMC13616327; doi:10.1002/advs.78005)
Supplement: Supplementary file 1 [file ADVS-9999-e78005-s001.docx]

**Supplementary Material**

**Transformer-based multitask framework integrating habitat and deep learning for predicting early disease control and survival in immunotherapy-treated hepatocellular carcinoma**

[Appendices	1](#_Toc238495432)

[Appendix 1. Study design and cohorts	1](#_Toc238495433)

[Appendix 2. mRECIST response assessment and central review	1](#_Toc238495434)

[Appendix 3. Image preprocessing and tumor segmentation	2](#_Toc238495435)

[Appendix 4. Subregion generation and feature extraction	3](#_Toc238495436)

[Appendix 5. Model construction	5](#_Toc238495437)

[Appendix 6. Multi-omics analysis	7](#_Toc238495438)

[Appendix 7. Digital pathology quantitative analysis	7](#_Toc238495439)

[Appendix 8. scRNA-seq analysis	8](#_Toc238495440)

[Supplementary Tables	10](#_Toc238495441)

[Table S1. Contrast-enhanced CT scan parameters from multiple centers	10](#_Toc238495442)

[Table S2. Radiomic features extracted for each voxel and their definitions	11](#_Toc238495443)

[Table S3. Description of features extracted from pathological images	12](#_Toc238495444)

[Table S4. Gene list of signatures	13](#_Toc238495445)

[Table S5. Tumor response in the training, internal validation, and external test sets	14](#_Toc238495446)

[Table S6. Baseline characteristics of patients from cohorts for biological interpretation	15](#_Toc238495447)

[Table S7. Agreement analysis for mRECIST-based early tumor response assessment in the central review subset	16](#_Toc238495448)

[Table S8. Univariate and multivariate analyses of logistic regression in the training set	17](#_Toc238495449)

[Table S9. Univariate and multivariate analysis of Cox regression in the training set	19](#_Toc238495450)

[Table S10. Correlation analyses between prediction models for early disease control in the training set	21](#_Toc238495451)

[Table S11. Correlation analyses between prediction models for early disease control in the internal validation set	21](#_Toc238495452)

[Table S12. Correlation analyses between prediction models for early disease control in the external test set	21](#_Toc238495453)

[Table S13. Correlation analyses between prediction models for overall survival in the training set	21](#_Toc238495454)

[Table S14. Correlation analyses between prediction models for overall survival in the internal validation set	21](#_Toc238495455)

[Table S15. Correlation analyses between prediction models for overall survival in the external test set	21](#_Toc238495456)

[Table S16. Ablation analyses of ECOS-Net on early disease control prediction across the sets	22](#_Toc238495457)

[Table S17. Ablation analyses of ECOS-Net on overall survival prediction across the sets	22](#_Toc238495458)

[Table S18. Tumor response between groups stratified by the ECOS-DC model in all sets	23](#_Toc238495459)

[Table S19. Multivariable logistic regression analyses of the ECOS-DC model and clinical factors on early disease control in the training, internal validation, and external test sets	24](#_Toc238495460)

[Table S20. Performance of the ECOS-DC model across subgroups	25](#_Toc238495461)

[Table S21. Multivariable Cox regression analyses of the ECOS-OS model and clinical factors on progression-free survival in the training, internal validation, and external test sets	27](#_Toc238495462)

[Table S22. Performance of ECOS-Net across CT acquisition subgroups	28](#_Toc238495463)

[Table S23. Comparison of molecular characteristics between groups in the ECOS-DC model	29](#_Toc238495464)

[Table S24. Comparison of molecular characteristics between groups in the ECOS-OS model	29](#_Toc238495465)

[Table S25. Comparison of quantitative pathological parameters between groups in the ECOS-DC model	30](#_Toc238495466)

[Table S26. Comparison of quantitative pathological parameters between groups in the ECOS-OS model	31](#_Toc238495467)

[Supplementary Figures and Figure legends	32](#_Toc238495468)

[Figure S1. Flowchart of exclusion criteria.	32](#_Toc238495469)

[Figure S2. Evaluation of the optimal number of clusters.	33](#_Toc238495470)

[Figure S3. Principal component analysis (PCA) of radiomic features before and after ComBat harmonization.	34](#_Toc238495471)

[Figure S4. Feature selection using LASSO regression.	35](#_Toc238495472)

[Figure S5. Calibration curve and decision curve analyses of the ECOS-DC, Clinical-DC, and Integrated-DC models.	36](#_Toc238495473)

[Figure S6. Heatmap of clinical features and different models’ predictions in the ICI cohort.	37](#_Toc238495474)

[Figure S7. Tumor response distribution in the lower and higher probability groups.	38](#_Toc238495475)

[Figure S8. Predicted probabilities in the lower and higher probability groups.	39](#_Toc238495476)

[Figure S9. Subgroup analyses of early disease control based on the ECOS-DC model.	40](#_Toc238495477)

[Figure S10. Kaplan-Meier curves of PFS in the training, internal validation, and external test sets.	41](#_Toc238495478)

[Figure S11. Calibration curves of the survival models.	42](#_Toc238495479)

[Figure S12. Decision curve analyses of the survival models.	43](#_Toc238495480)

[Figure S13. Subgroup analyses of overall survival based on the ECOS-OS model.	44](#_Toc238495481)

[Figure S14. Subgroup analyses of progression-free survival based on the ECOS-OS model.	45](#_Toc238495482)

[Figure S15. Subgroup analyses of overall survival based on the Integrated-OS model.	46](#_Toc238495483)

[Figure S16. Subgroup analyses of progression-free survival based on the Integrated-OS model.	47](#_Toc238495484)

[Figure S17. Biological pathway analyses of ECOS-DC and ECOS-OS models.	48](#_Toc238495485)

[Figure S18. Biological differences of ECOS-DC and ECOS-OS models.	49](#_Toc238495486)

[Figure S19. Immune checkpoint gene expression and mutational landscape.	50](#_Toc238495487)

[Figure S20. The cell annotation genes for 10 cell types in scRNA-seq analysis.	51](#_Toc238495488)

[Figure S21. scRNA-seq analyses between higher probability-lower risk and lower probability-higher risk groups.	52](#_Toc238495489)

# Appendices

## Appendix 1. Study design and cohorts

This study utilized four cohorts: an ICI cohort for model development and validation, and three cohorts for exploratory biological interpretation: (1) TCGA-LIHC cohort, (2) whole-slide image (WSI) cohort, and (3) single-cell RNA sequencing (scRNA-seq) cohort. The flowchart of patient inclusion and exclusion is presented in Figure S1.

(1) ICI cohort (model development and validation): The ICI cohort retrospectively enrolled 832 HCC patients treated with ICIs from seven institutions: WHUH, HCH, APH, HBCH, XYH, SAHMU, and TFCH. Among these, 652 patients from WHUH, HCH, and APH were randomly split (7:3) into a training set (n = 456) and an internal validation set (n = 196). An external test set comprised 180 patients from four additional independent hospitals and was used to evaluate external model performance.

Inclusion criteria were: (a) HCC diagnosis confirmed by histopathology or according to the European Association for the Study of the Liver (EASL) criteria; (b) Child-Pugh A or B; (c) receipt of first-line ICI therapy; (d) age >18 years; and (e) contrast-enhanced abdominal CT performed within 2 weeks prior to the first ICI therapy. Exclusion criteria were: (a) presence of other malignancies; (b) Child-Pugh C; (c) BCLC stage A; (d) poor-quality contrast-enhanced CT images; and (e) prior local therapy before initiation of ICIs.

(2) TCGA-LIHC cohort: To explore potential associations between imaging model outputs and molecular features, we rigorously screened the TCGA/TCIA database for HCC patients with concurrent availability of contrast-enhanced abdominal CT scans, clinical information, transcriptomic data, somatic mutation data, and matched whole-slide images (WSI). A final set of 39 patients met all criteria and underwent multi-omics analysis.
(3) WSI cohort: This cohort allowed us to explore the spatial distribution of the tumor microenvironment and to examine potential intergroup differences among patients stratified by ECOS-Net predictions. It comprised 396 HCC patients, including the aforementioned 39 matched TCGA-LIHC samples and an additional 357 surgical resection HCC patients from two independent institutions (APH and SRRSH).

(4) scRNA-seq cohort: This cohort provided an opportunity to explore potential cellular composition and functional states that could be associated with ECOS-Net predictions. It included a total of 6 treatment-naive HCC patients who underwent surgical resection. All patients had contrast-enhanced abdominal CT scans before surgery.

## Appendix 2. mRECIST response assessment and central review

Tumor response was evaluated according to HCC modified RECIST (mRECIST) [1]. Target lesion response was assessed based on the sum of the longest diameters of viable enhancing tumor tissue in the arterial phase. Complete response (CR) was defined as the disappearance of any intratumoral arterial enhancement in all target lesions. Partial response (PR) was defined as at least a 30% decrease in the sum of the diameters of viable enhancing target lesions, taking the baseline sum as the reference. Progressive disease (PD) was defined as at least a 20% increase in the sum of the diameters of viable enhancing target lesions, taking the smallest sum recorded since treatment initiation as the reference, or the appearance of new lesions. Stable disease (SD) was defined as neither sufficient shrinkage to qualify for PR nor sufficient increase to qualify for PD. Non-target lesion response and new lesions were also considered according to mRECIST to determine patient-level overall response. Early disease control (DC) was defined as CR, PR, or SD at the first follow-up CT performed 4–8 weeks after ICI initiation.

The primary mRECIST response assessment used for endpoint definition was performed locally at each participating center according to uniform HCC mRECIST criteria. The original local assessors were blinded to subsequent clinical outcomes and ECOS-Net predictions. To evaluate the reliability of the local assessments, a post hoc central blinded review was performed in a stratified random subset of 150 patients from the ICI cohort. Patients were randomly sampled after stratification by participating center and original local mRECIST response category. Baseline CT images and the first follow-up CT images obtained 4–8 weeks after ICI initiation were independently reviewed by two radiologists from the coordinating center (Wuhan Union Hospital), G.L.Z. and S.S.J., with 4 and 3 years of experience in abdominal radiology, respectively, according to HCC mRECIST criteria. The two central reviewers were blinded to the original local response assessments, ECOS-Net predictions, and subsequent survival outcomes. Disagreements between the two central reviewers were resolved by a senior abdominal radiologist, L.Y., with 27 years of experience, to generate the final central review results. Cohen’s kappa coefficient (κ) was used to evaluate inter-reader agreement between the two central reviewers and concordance between the original local assessments and the final central review results.

## Appendix 3. Image preprocessing and tumor segmentation

Arterial-phase contrast-enhanced CT images were uniformly selected for subsequent analysis. This phase was chosen because HCC typically demonstrates arterial-phase enhancement, providing favorable tumor-liver contrast for segmentation. Arterial-phase enhancement reflects tumor angiogenesis, perfusion, and viable tumor components, providing biologically relevant imaging information. In addition, mRECIST-based tumor response assessment primarily focuses on viable arterial-enhancing tumor components; therefore, arterial-phase feature extraction was consistent with the endpoint definition. Using a single routinely acquired phase also reduced additional variability related to multiphasic timing differences and phase matching across centers. All DICOM files were anonymized and converted to the NIfTI format.

Contrast-enhanced CT scans were performed using an intravenously administered iodine-based contrast agent (concentration: 300–350 mgI/mL) at a dosage of 1.3–1.5 mL/kg and an injection rate of 2.0–3.0 mL/s. A summary of the detailed imaging protocols from each participating center is available in Table S1. To ensure consistency across patients, standardized preprocessing was applied to CT images: (1) window-level adjustment was performed using standard abdominal settings (window width: 500 Hounsfield Units (HU), window level: 50 HU); and (2) B-spline interpolation was used to resample voxels into isotropic 1 mm × 1 mm × 1 mm spacing. These preprocessing steps were implemented to mitigate inter-scanner variability, enhance lesion conspicuity, and improve the robustness and reproducibility of subsequent feature extraction.

Tumor volumes of interest (VOIs) were delineated using a semi-automated segmentation pipeline based on the nnU-Net framework supplemented by manual correction. A pre-trained nnU-Net model generated an initial tumor mask. This model was developed using an independent arterial-phase CT segmentation dataset of 261 HCC patients from Wuhan Union Hospital and achieved a Dice coefficient of 0.823. Patient identifiers, imaging dates, and examination numbers were checked to confirm that this segmentation dataset had no patient-level or lesion-level overlap with the ICI, TCGA-LIHC, WSI, or scRNA-seq cohorts. Importantly, the nnU-Net outputs were used only as initial masks and were not directly used as the final VOIs for feature extraction. Two radiologists (G.L.Z. and S.S.J., with 4 and 3 years of experience in abdominal radiology, respectively) then independently reviewed and manually corrected the contours slice-by-slice using ITK-SNAP software (version 3.8; https://www.itksnap.org). Both reviewers were blinded to clinical outcomes and model predictions during this process. In cases of disagreement between the two radiologists, a third senior abdominal radiologist (L.Y., with 27 years of experience) served as the adjudicator to provide the final segmentation. In a randomly selected subset of 150 patients, the manually corrected VOIs from the two radiologists showed excellent agreement. The Dice coefficient was 0.921, the 95th percentile Hausdorff distance (HD95) was 3.79 mm, and the tumor volume intraclass correlation coefficient (ICC) was 0.974 (95% CI: 0.963–0.981), indicating high spatial overlap, small boundary variation, and excellent volumetric consistency between readers.

## Appendix 4. Subregion generation and feature extraction

**Appendix 4.1. Habitat subregion generation and feature extraction**

To characterize intratumoral heterogeneity (ITH), we employed a sliding window approach (3 × 3 × 3 voxels) to extract detailed voxel-level features from the VOIs. This strategy enhanced the delineation of tumor subregions and the characterization of heterogeneity. For patients with multifocal tumors, we selected the lesion with the maximal diameter on arterial-phase CT as the target lesion. In accordance with the PyRadiomics documentation [2], the specific feature definitions are detailed in Table S2.

Subsequently, the K-means algorithm was applied to cluster the feature vectors of all voxels within the VOI, thereby partitioning the tumor into distinct habitat subregions reflecting varying microenvironmental characteristics. To determine the optimal number of clusters (k), we tested k values from 3 to 9 to balance the capture of heterogeneity and the avoidance of over-fragmentation. The optimal k was selected based on a joint evaluation of the Calinski-Harabasz (CH) index and the Davies-Bouldin (DB) index. Ultimately, k = 3 was selected as the optimal number of clusters, corresponding to the maximum CH index and the minimum DB index (Figure S2).

Radiomic features were extracted using PyRadiomics (v3.0.1), including shape (n = 14), first-order intensity (n = 18), and texture (n = 73) features. Texture features comprised gray-level co-occurrence matrix (GLCM, n = 22), gray-level dependence matrix (GLDM, n = 14), gray-level run-length matrix (GLRLM, n = 16), gray-level size zone matrix (GLSZM, n = 16), and neighboring gray-tone difference matrix (NGTDM, n = 5) features to quantify spatial dependency patterns. For each habitat subregion, radiomic features were extracted from original images and filtered transformations (e.g., exponential and wavelet), yielding 1,288 features per subregion. Subregion features were concatenated using an early-fusion strategy, resulting in 3,864 features. To mitigate inter-center batch effects, ComBat harmonization was applied (Figure S3). Features were then z-score standardized to a mean of 0 and a standard deviation of 1. A systematic feature selection pipeline was performed: (1) the Mann-Whitney U test was used to retain features with *P* < 0.05, resulting in 1,299 features; (2) Spearman correlation analysis (|r| > 0.9) was used to remove redundant features, yielding 244 features; and (3) least absolute shrinkage and selection operator (LASSO) regression was employed to construct the optimal feature subset, leveraging its inherent regularization to eliminate features with low predictive value, ultimately retaining 16 features. The penalty parameter (λ) was determined via 10-fold cross-validation to minimize the risk of overfitting (Figure S4). The parameters for habitat feature standardization and selection were derived exclusively from the training set and then fixed when applied to the internal validation and external test sets.

**Appendix 4.2. Multi-channel 2.5D deep learning feature extraction**

To minimize potential interference from surrounding liver parenchyma and vascular structures, this study employed an ROI-only strategy to isolate the tumor region for deep learning (DL) feature extraction. For each patient, including patients with multifocal tumors, the measurable intrahepatic HCC lesion with the maximal diameter on baseline arterial-phase CT was selected as the target lesion. Within this target lesion, the axial, sagittal, and coronal slices with the largest tumor cross-sectional area were selected separately. These three orthogonal 2D tumor images were combined as a three-channel 2.5D input image and resized to 256 × 256 pixels. This strategy captured representative morphological and textural information from three orthogonal views. For multifocal cases, the same slice selection and feature extraction strategy was applied to the selected target lesion.

We adopted a transfer learning strategy by initializing the DenseNet121 backbone with ImageNet-pretrained weights. The model employed the Adam optimizer with a learning rate of 0.0001 and a batch size of 64. The 2.5D DL features were extracted from the global average pooling layer of DenseNet121. To reduce dimensionality and facilitate subsequent model integration, principal component analysis (PCA) was applied to reduce the extracted feature vectors to 32 principal components, which served as the final 2.5D DL features for the downstream modeling framework. Because one target lesion was selected for each patient, the lesion-level 2.5D DL features were used as patient-level features for ECOS-Net prediction.

## Appendix 5. Model construction

**Appendix 5.1. Early control and overall survival network (ECOS-Net)**

We constructed a transformer-based multitask framework named ECOS-Net (Figure 2), designed to predict early disease control (DC) and overall survival (OS) simultaneously in patients with HCC receiving ICI therapy. The network architecture consisted of three core components: (1) a dual-branch feature encoder, (2) a transformer-based feature fusion module, and (3) task-specific output heads. Specifically, habitat features and 2.5D DL features were fed into two parallel encoders (constructed by stacking multilayer fully connected blocks) to obtain embedded representations. The embeddings from both branches were then concatenated and fed into a transformer-based multi-head attention module to achieve cross-branch feature interaction and fusion. Finally, mean pooling was applied to the transformer output to obtain sample-level global representations, which were separately fed into a DC classification head and an OS prediction head, yielding the ECOS-DC and ECOS-OS outputs. The transformer module consisted of two transformer layers and eight attention heads, with an embedding dimension of 48. The habitat branch used fully connected layers with hidden dimensions of 64, 32, and 32 and a dropout rate of 0.3, whereas the 2.5D DL branch used hidden dimensions of 256, 128, and 64 and a dropout rate of 0.3.

The model was trained using the Adam optimizer with an initial learning rate of 0.001, no weight decay, and a batch size of 16. The model was trained for 50 epochs. Conventional early stopping was not applied; instead, the checkpoint with the best validation multitask performance was retained for final evaluation, avoiding reliance on the final-epoch checkpoint alone. The multitask loss was calculated as the weighted sum of the early DC classification loss and the OS Cox loss, with equal task weights of 0.5 and 0.5, respectively. The development cohort was randomly split into training and internal validation sets at a 7:3 ratio, which was kept fixed throughout model development, whereas the external test set was used only for independent evaluation. Model parameters were initialized using the default random initialization strategy provided by the DL framework. ECOS-Net is described in detail below:

Multi-Head Attention Mechanism: A transformer-based model was employed for feature fusion to improve task-specific predictive performance. The proposed model consisted of three key components: transformer encoding, feature fusion, and task prediction heads.

1. Transformer Encoding: Each modality is first encoded by an MLP-based branch encoder (three ReLU-activated hidden layers: 64-32-32 and 256-128-64 units for the branches with input dimensions of 16 and 32, respectively) to obtain a latent embedding with dimensionality $\text{d}$ =48. The input feature sequence $\mathbf{X}\in{\mathbb{\mathbb{R}}}^{n\times d}$, where $\text{n}$ represents the number of feature branches (tokens) and $\text{d}$ their embedding dimensionality was first transformed into query ($\text{Q}$), key ($\text{K}$), and value ($\text{V}$) matrices:

$$\mathbf{Q}=\mathbf{X}\mathbf{W}_{Q}, \mathbf{K}=\mathbf{X}\mathbf{W}_{K}, \mathbf{V}=\mathbf{X}\mathbf{W}_{V}$$

where $\text{W}_{\text{Q}}\text{,}\text{W}_{\text{K}}\text{,}\text{W}_{\text{V}}\text{∈}\text{R}^{\text{d}\text{×}\text{d}_{\text{k}}}$ are learnable projection matrices. The multi-head self-attention mechanism was then applied, computing attention output using the scaled dot-product attention:

$$\text{Attention}\left( \mathbf{Q},\mathbf{K},\mathbf{V} \right)=\text{softmax}\left( \frac{\mathbf{Q}\mathbf{K}^{T}}{\sqrt{d_{k}}} \right)\mathbf{V}$$

where $\sqrt{\text{d}_{\text{k}}}$ serves as a scaling factor to stabilize gradients. This mechanism allows the model to dynamically prioritize the most relevant features while capturing complex interactions across different modalities.

2. Feature Fusion: The outputs from multiple attention heads were concatenated and passed through a feed-forward network (FFN) with GELU activation:

$$\text{FFN}\left( \text{X} \right)\text{=GELU}\left( \text{W}_{\text{1}}\text{X}\text{+}\text{b}_{\text{1}} \right)\text{W}_{\text{2}}\text{+}\text{b}_{\text{2}}$$

where $\text{W}_{\text{1}}\text{,}\text{W}_{\text{2}}$ are weight matrices and $\text{b}_{\text{1}}\text{,}\text{b}_{\text{2}}$ are bias terms.

This step refines the representation by capturing both local and global dependencies among features. A residual connection with layer normalization was applied to stabilize training. After the Transformer encoder, we apply mean pooling over tokens to obtain a sample-level representation:

$$\begin{matrix} z=\frac{1}{n}\sum_{i=1}^{n} H_{i} \end{matrix}$$

where $H$ denotes the fused token representations from the Transformer encoder and $H_{i}$ is the representation of the $i$-th token.

3. Prediction Layer: The fused feature representation was subsequently fed into a classification head and a prognostic head to predict disease control and overall survival. For classification, the final output probability $\hat{\text{y}}$ for disease control was obtained through a fully connected layer followed by a softmax activation:

$$\hat{y}=\text{softmax}\left( \mathbf{W}_{o}\mathbf{z}+b_{o} \right)$$

where $\text{z}$ represents the pooled sample-level representation, and $\text{W}_{\text{o}}\text{,}\text{b}_{\text{o}}$ are learnable parameters.

Survival prediction was performed using the Cox proportional hazards loss. The model was trained to minimize the negative partial log-likelihood (Cox loss), which measures the agreement between predicted risks and observed survival times.

$$\mathcal{\mathcal{L}}(\theta)=-\frac{1}{N_{E}}\sum_{i:\delta_{i}=1} \left( h_{\theta}(x_{i})-\log\sum_{j\in R(T_{i})} e^{h_{\theta}(x_{j})} \right)$$

where $h_{\theta}(x_{i})$ represents predicted log-risk for subject $i$; $R(T_{i})$ represents subjects at risk at time $T_{i}$; $N_{E}$ is the number of observed events.

**Appendix 5.2. Clinical and Integrated Models**

Within the training set, we first employed univariate logistic regression to identify clinical variables associated with early DC. Variables with *P* < 0.1 were included in a subsequent multivariable logistic regression model, and those retaining significance (*P* < 0.05) in the multivariable analysis were used to construct the Clinical-DC model. A similar procedure was followed for OS. Variables with *P* < 0.1 in univariate Cox proportional hazards regression were entered into a multivariable Cox model, and significant variables (*P* < 0.05) were retained to build the Clinical-OS model.

To leverage the complementary information from both the imaging and clinical models, we further integrated the predictions from these clinical models with the corresponding outputs from ECOS-Net. This integration yielded the Integrated-DC and Integrated-OS models for the comprehensive prediction of early DC and OS, respectively.

## Appendix 6. Multi-omics analysis

Multi-omics analysis was performed using TCGA-LIHC data. Differential expression between ECOS-Net-defined groups was assessed using the “limma” R package. Given the limited sample size of the TCGA-LIHC cohort, genes with |log2(fold change)| >1 and *P* value < 0.05 were defined as nominally differentially expressed genes (DEGs) and were used only for exploratory downstream analyses. Gene Ontology (GO) and Kyoto Encyclopedia of Genes and Genomes (KEGG) analyses were performed using the nominal DEGs, with enriched terms considered statistically significant at Benjamini-Hochberg adjusted *P <* 0.05. Gene set enrichment analysis (GSEA) was performed using a ranked list of all genes with KEGG and Reactome gene sets utilizing the “clusterProfiler” package, with pathway-level significance assessed using Benjamini-Hochberg adjusted *P* value < 0.05.

The relative abundances of immune and stromal cell components in tumor tissue were quantitatively estimated using the MCP-counter algorithm. Enrichment scores for immune cell-associated gene signatures were calculated via single-sample gene set enrichment analysis (ssGSEA). To comprehensively assess the tumor microenvironment composition, we employed the ESTIMATE algorithm to compute StromalScore, ImmuneScore, and ESTIMATEScore for each sample.

Furthermore, we analyzed differences in established biomarkers across the stratified groups. These analyses included comparisons of CD274 (PD-L1) expression levels, microsatellite instability (MSI), and tumor mutational burden (TMB). Somatic mutation profiles were processed with the “maftools” package to visualize the top 30 mutated genes and to calculate TMB. MSI values were derived from an established online resource [3]. Finally, to evaluate the association between imaging model outputs and inflammatory/immune scores, we incorporated immune-related gene signatures as defined by Thorsson et al. [4] in their pan-cancer analysis of TCGA. For the molecular and immune features analyzed in this study, *P* values from group comparisons were adjusted using the Benjamini-Hochberg method. Both nominal and adjusted *P* values were reported, and an adjusted *P* value < 0.05 was considered statistically significant.

## Appendix 7. Digital pathology quantitative analysis

A pathologist specializing in abdominal oncology selected the most representative tumor slide from hematoxylin and eosin (H&E)-stained whole-slide image (WSI) for each patient and delineated the tumor regions using QuPath software (https://qupath.github.io). Subsequently, a DL pipeline based on the CellViT framework [5] (https://github.com/TIO-IKIM/CellViT) was applied to the annotated regions for automated nucleus segmentation and classification. Nuclei were categorized into five classes: neoplastic cells, inflammatory cells, epithelial cells, dead cells, and connective cells.

To further quantify the spatial distribution characteristics of these cells within the tumor region, we developed customized Groovy scripts within the QuPath platform. These scripts were designed to automatically extract spatial pathology metrics, including the densities of different cell types, the distance from each neoplastic cell to its nearest inflammatory cell, clustering features, and tumor-immune colocalization. Detailed definitions and descriptions of the specific metrics are provided in Table S3. Both nominal *P* values and Benjamini-Hochberg adjusted *P* values were reported in Tables S25 and S26. An adjusted *P* value < 0.05 was considered statistically significant, whereas findings based only on nominal *P* values were interpreted as exploratory trends.

## Appendix 8. scRNA-seq analysis

The tissue samples used in this analysis were derived from surgical resection specimens from patients with HCC who had not received prior ablation therapy, chemotherapy, or radiotherapy. Single-cell suspensions were prepared from tissue samples via rapid enzymatic digestion, and sequencing was performed on the DNBelab C4 platform. scRNA-seq data from six samples were analyzed using Seurat (v5.1.0). Quality control was performed using the following criteria: (1) 500-6,000 detected genes per cell; (2) 1,000-30,000 unique molecular identifiers (UMI) per cell; (3) mitochondrial gene proportion < 15%; and (4) hemoglobin gene proportion < 1%. The data were normalized using the LogNormalize method with a scale factor set to 10,000. Highly variable genes were selected by the FindVariableFeatures function, with the top 2,000 genes for subsequent analysis. Data scaling was performed using the ScaleData function. Principal component analysis (PCA) was conducted based on the selected genes, and the top 15 principal components were used for downstream steps. Batch effects were corrected using the Harmony package, followed by cell clustering via the FindNeighbors and FindClusters functions based on the first 15 principal components. The clustering results were visualized using uniform manifold approximation and projection (UMAP). Cell types were annotated according to classical marker genes and relevant literature. DEGs within each cluster were identified using the FindMarkers function and visualized in volcano plots. GSEA was further performed based on the Hallmark gene sets to identify biological pathways significantly associated with specific cell states or functions. Additionally, immune-related gene signatures from existing studies were used with the AddModuleScore function to calculate signature scores for specific cellular functions [6-9], including cytotoxicity, Treg activity, M1/M2 polarization, and dendritic cell maturation. The relevant gene signatures are detailed in Table S4. *P* values derived from differential expression analyses within each cell type, GSEA, and comparisons of immune-related signature scores were adjusted using the Benjamini-Hochberg method, and adjusted *P <* 0.05 was considered statistically significant. Given the limited number of scRNA-seq samples, these cell-level findings should be interpreted as exploratory.

1.	R. Lencioni and J. M. Llovet, “Modified RECIST (mRECIST) assessment for hepatocellular carcinoma,” *Seminars in Liver Disease* 30, no. 1 (2010): 52–60, <https://doi.org/10.1055/s-0030-1247132>.

2.	J. J. M. van Griethuysen, A. Fedorov, C. Parmar, et al., “Computational Radiomics System to Decode the Radiographic Phenotype,” *Cancer Research* 77, no. 21 (2017): e104–e107, https://doi.org/10.1158/0008-5472.CAN-17-0339.

3.	D. Tang, M. Chen, X. Huang, et al., “SRplot: A free online platform for data visualization and graphing,” *PLoS One* 18, no. 11 (2023): e0294236, <https://doi.org/10.1371/journal.pone.0294236>.

4.	V. Thorsson, D. L. Gibbs, S. D. Brown, et al., “The Immune Landscape of Cancer,” *Immunity* 48, no. 4 (2018): 812–830.e14, <https://doi.org/10.1016/j.immuni.2018.03.023>.

5.	F. Hörst, M. Rempe, L. Heine, et al., “CellViT: Vision Transformers for precise cell segmentation and classification,” *Medical Image Analysis* 94 (2024): 103143, <https://doi.org/10.1016/j.media.2024.103143>.

6.	N. Ding, Y. Xie, Y. Guo, et al., “Dynamic Single-Cell Profiling Reveals Novel Immune Regulatory Mechanism of ITK Inhibitor Soquelitinib in Refractory T Cell Lymphoma,” *Blood* 142, Supplement 1 (2023): 1442, https://doi.org/10.1182/blood-2023-174421.

7.	S. Sakaguchi, N. Mikami, J. B. Wing, A. Tanaka, K. Ichiyama, and N. Ohkura, “Regulatory T Cells and Human Disease,” *Annual Review of Immunology* 38 (2020): 541–566, <https://doi.org/10.1146/annurev-immunol-042718-041717>.

8.	M. Rakina, I. Larionova, and J. Kzhyshkowska, “Macrophage diversity in human cancers: New insight provided by single-cell resolution and spatial context,” *Heliyon* 10, no. 7 (2024): e28332, <https://doi.org/10.1016/j.heliyon.2024.e28332>.

9.	J. Banchereau and R. M. Steinman, “Dendritic cells and the control of immunity,” *Nature* 392, no. 6673 (1998): 245–252, <https://doi.org/10.1038/32588>.

# Supplementary Tables

## Table S1. Contrast-enhanced CT scan parameters from multiple centers

| Parameter | WHUH | HCH | APH | XYH | HBCH | SAHMU | TFCH | SRRSH |
| --- | --- | --- | --- | --- | --- | --- | --- | --- |
| CT scanners | Siemens, Philips, Toshiba, GE | Siemens, United Imaging | GE | Siemens | Siemens, GE | Siemens | Siemens, GE | Siemens, GE |
| Slice thickness | 0.625–5 mm | 0.625–1.5 mm | 0.625–5 mm | 1.5 mm | 0.625–5 mm | 5 mm | 1–1.5 mm | 5–7 mm |
| Slice interval | 1–1.5 mm | 1.5 mm | 1.5 mm | 1.5 mm | 1.5 mm | 1.5 mm | 0.8–1.3 mm | 5–7 mm |
| Detector rows | 64, 128, 256 | 64, 128 | 64 | 128 | 64 | 192 | 128 | 64, 96 |
| Tube current | Auto mA | Auto mA | 250–350 mA | Auto mA | Auto mA | Auto mA | Auto mA | 180–260 mA |
| Tube voltage | 70–140 kV | 120–130 kV | 120 kV | 120 kV | 120 kV | 120 kV | 120 kV | 120 kV |
| Data matrix | 512 × 512 | 512 × 512 | 512 × 512 | 512 × 512 | 512 × 512 | 512 × 512 | 512 × 512 | 512 × 512 |
| Helical pitch | 0.5–1.5 | 0.6–1 | 1.375 | 0.8 | 0.5–1.5 | 0.8 | 0.9–1 | 0.5–1.5 |

Abbreviations: WHUH, Union Hospital, Tongji Medical College, Huazhong University of Science and Technology; HCH, Henan Cancer Hospital; APH, Anhui Provincial Hospital; HBCH, Hubei Cancer Hospital; XYH, Xiangya Hospital, Central South University; SAHMU, The Second Affiliated Hospital of Anhui Medical University; TFCH, Tianjin First Central Hospital; SRRSH, Sir Run Run Shaw Hospital, Zhejiang University School of Medicine.

## Table S2. Radiomic features extracted for each voxel and their definitions

| Radiomic Features | Definitions |
| --- | --- |
| firstorder_Entropy | Entropy quantifies the uncertainty or randomness in image values, reflecting the average information needed to encode these values. |
| firstorder_Median | The median gray level intensity within the ROI |
| firstorder_MeanAbsoluteDeviation | The average distance of all intensity values from the mean value of the image array. |
| glcm_DifferenceAverage | A measure of the relationship between the frequency of pairs with similar intensity values and those with differing intensity values. |
| glcm_DifferenceEntropy | A measure of the randomness or variability in the differences of neighborhood intensity values. |
| glcm_DifferenceVariance | A measure of heterogeneity that gives greater weight to pairs with differing intensity levels that deviate more from the mean. |
| glcm_Imc1 | Quantifies the correlation between pixel pairs using joint and marginal entropy. Values close to 1 indicate strong correlation. |
| glcm_Imc2 | A modified version of IMC1 that accounts for the uncertainty in the GLCM distribution. |
| glcm_InverseVariance | A weighted average of the inverse of squared intensity differences in the GLCM, highlighting small differences between pixel pairs. |
| glcm_JointEnergy | A measure of homogeneous patterns in the image. Higher energy indicates a greater frequency of neighboring intensity value pairs. |
| glcm_JointEntropy | A measure of the randomness or variability in the neighborhood intensity values. |
| glcm_SumEntropy | A measure of the sum of neighborhood intensity value differences. |
| glrlm_LongRunEmphasis | A measure of the distribution of long run lengths, where higher values indicate longer runs and coarser structural textures |
| glrlm_RunEntropy | A measure of the uncertainty or randomness in the distribution of run lengths and gray levels. Higher values indicate greater heterogeneity in texture patterns. |
| glrlm_RunVariance | A measure of the variance in run lengths. |
| glszm_SizeZoneNonUniformityNormalized | A measure of the variability in size zone volumes across the image, with lower values indicating greater homogeneity in zone size volumes. |
| glszm_SmallAreaHighGrayLevelEmphasis | A measure of the proportion of smaller size zones with higher gray-level values in the image. |
| ngtdm_Contrast | A measure of spatial intensity change, dependent on the overall gray level dynamic range. High contrast occurs when both the dynamic range and spatial change rate are large, indicating an image with a broad gray level range and significant changes between voxels and their neighbors. |
| ngtdm_Strength | A measure of the prominence of primitives in an image. Its value is high when the primitives are clearly defined and visible, such as in an image with gradual intensity changes but large coarse differences in gray level intensities. |

The definition of features is consistent with the PyRadiomics documentation. Abbreviations: GLCM, Gray-Level Co-occurrence Matrix; IMC, Informational Measure of Correlation; GLRLM, Gray-Level Run-Length Matrix; GLSZM, Gray-Level Size-Zone Matrix; NGTDM, Neighborhood Gray-Tone Difference Matrix.

## Table S3. Description of features extracted from pathological images

| Feature Name | Description |
| --- | --- |
| Density of neoplastic cells | Density of neoplastic cells within the tumor area (cells/mm²). |
| Density of inflammatory cells | Density of inflammatory cells within the tumor area (cells/mm²). |
| Density of connective cells | Density of connective (stromal) cells within the tumor area (cells/mm²). |
| Density of dead cells | Density of dead cells within the tumor area (cells/mm²). |
| Tumor cellularity | A composite metric defined as neoplastic cell density × tumor maximum diameter. |
| Avg Tumor-Inflammatory Distance | Average distance between each tumor cell and its nearest inflammatory cell (µm). |
| Min Tumor-Inflammatory Distance | Minimum observed distance between tumor and inflammatory cells (µm). |
| Max Tumor-Inflammatory Distance | Maximum observed distance between tumor and inflammatory cells (µm). |
| Ripley’s K (Immune) | Ripley’s K-function value quantifying the spatial clustering of inflammatory cells. |
| Tumor-Immune Colocalization | Proportion of tumor cells located within 50 µm of an inflammatory cell. |

## Table S4. Gene list of signatures

| Cytotoxicity | Treg activity | M1 polarization | M2 polarization | DC maturation |
| --- | --- | --- | --- | --- |
| GZMA | CTLA4 | IL1B | IL10 | CD80 |
| GZMK | TIGIT | TNF | MARCO | CD86 |
| PRF1 | FOXP3 | CD80 | MRC1 | CD83 |
| GZMH | TGFB1 | CXCL9 | CD163 | CD40 |
| NKG7 | NT5E | CXCL10 | IL1R2 | IL12B |
| CTSW | LGALS1 | CD86 | IL4R | IL10 |
| GZMB | IL2RA |  |  | TNF |

Abbreviations: DC, dendritic cell.

## Table S5. Tumor response in the training, internal validation, and external test sets

|  | Training set  (n = 456) | Internal validation set  (n = 196) | External test set  (n = 180) | *P* value |
| --- | --- | --- | --- | --- |
| Tumor response, n (%) |  |  |  | 0.307 |
| CR | 0 (0%) | 0 (0%) | 0 (0%) |  |
| PR | 110 (24.1%) | 62 (31.6%) | 53 (29.4%) |  |
| PD | 147 (32.2%) | 54 (27.6%) | 52 (28.9%) |  |
| SD | 199 (43.6%) | 80 (40.8%) | 75 (41.7%) |  |
| ORR, n (%) | 110 (24.1%) | 62 (31.6%) | 53 (29.4%) | 0.101 |
| DCR, n (%) | 309 (67.8%) | 142 (72.4%) | 128 (71.1%) | 0.433 |

Abbreviations: CR, complete response; PR, partial response; SD, stable disease; PD, progressive disease; ORR, objective response rate; DCR, disease control rate.

## Table S6. Baseline characteristics of patients from cohorts for biological interpretation

| Characteristics | APH (n = 277) | SRRSH (n = 80) | TCGA-LIHC (n = 39) | scRNA-seq (n = 6) |
| --- | --- | --- | --- | --- |
| Age (years), median (IQR) | 58 (49, 66) | 62 (53, 69) | 64 (53, 68.5) | 61 (57.3, 67.8) |
| Sex, n (%) |  |  |  |  |
| Female | 36 (13%) | 12 (15%) | 15 (38.5%) | 0 (0%) |
| Male | 241 (87%) | 68 (85%) | 24 (61.5%) | 6 (100%) |
| Tumor number, n (%) |  |  |  |  |
| ≤ 3 | 275 (99.3%) | 80 (100%) | 34 (87.2%) | 4 (66.7%) |
| > 3 | 2 (0.7%) | 0 (0%) | 5 (12.8%) | 2 (33.3%) |
| Tumor diameter (cm), median (IQR) | 3 (2, 4) | 4.35 (3, 6) | 6.4 (3.3, 11.15) | 7.5 (6.6, 9) |
| HBV infection, n (%) |  |  |  |  |
| No | 85 (30.7%) | 15 (18.8%) | 26 (66.7%) | 0 (0%) |
| Yes | 192 (69.3%) | 65 (81.2%) | 13 (33.3%) | 6 (100%) |
| Albumin (g/L), median (IQR) | 41.4 (37.8, 43.9) | 41.05 (37.45, 44.4) | 39 (33, 42) | 39.3 (35.7, 40) |
| Child-Pugh, n (%) |  |  |  |  |
| A | 256 (92.4%) | 74 (92.5%) | 35 (89.7%) | 6 (100%) |
| B | 21 (7.6%) | 6 (7.5%) | 4 (10.3%) | 0 (0%) |
| AFP level (ng/mL), n (%) |  |  |  |  |
| < 400 | 171 (61.7%) | 58 (72.5%) | 30 (76.9%) | 6 (100%) |
| ≥ 400 | 106 (38.3%) | 22 (27.5%) | 9 (23.1%) | 0 (0%) |

Abbreviations: APH, Anhui Provincial Hospital; SRRSH, Sir Run Run Shaw Hospital, Zhejiang University School of Medicine; HBV, hepatitis B virus; AFP, alpha-fetoprotein; IQR, interquartile range.

## Table S7. Agreement analysis for mRECIST-based early tumor response assessment in the central review subset

| Comparison | Early tumor response | Cohen’s κ (95% CI) |
| --- | --- | --- |
| Between two central reviewers | CR/PR/SD/PD | 0.919 (0.857–0.968) |
| Between two central reviewers | DC/non-DC | 0.923 (0.840–0.984) |
| Original local assessment vs. final central review | CR/PR/SD/PD | 0.919 (0.859–0.969) |
| Original local assessment vs. final central review | DC/non-DC | 0.922 (0.843–0.984) |

Abbreviations: CR, complete response; PR, partial response; SD, stable disease; PD, progressive disease; DC, disease control. Cohen’s kappa coefficient (κ) was used to assess agreement.

## Table S8. Univariate and multivariate analyses of logistic regression in the training set

| Characteristics | Univariate analysis | | Multivariate analysis | |
| --- | --- | --- | --- | --- |
|  | Odds Ratio (95% CI) | *P* value | Odds Ratio (95% CI) | *P* value |
| Age (years) |  |  |  |  |
| < 60 | Reference |  |  |  |
| ≥ 60 | 1.034 (0.696–1.538) | 0.867 |  |  |
| Sex |  |  |  |  |
| Female | Reference |  |  |  |
| Male | 0.917 (0.537–1.566) | 0.750 |  |  |
| BMI (kg/m^2^) | 1.007 (0.948–1.070) | 0.818 |  |  |
| Types of ICIs |  |  |  |  |
| PD-1 | Reference |  |  |  |
| PD-L1 | 1.896 (0.755–4.763) | 0.173 |  |  |
| PD-1/CTLA-4 | 1.484 (0.153–14.397) | 0.733 |  |  |
| Treatment |  |  |  |  |
| ICIs alone | Reference |  |  |  |
| ICIs + TKIs | 1.137 (0.635–2.034) | 0.666 |  |  |
| ICIs + anti-VEGF | 1.447 (0.690–3.011) | 0.323 |  |  |
| HBV infection |  |  |  |  |
| No | Reference |  |  |  |
| Yes | 1.194 (0.665–2.145) | 0.553 |  |  |
| Cirrhosis, n (%) |  |  |  |  |
| No | Reference |  | Reference |  |
| Yes | 0.646 (0.408–1.025) | 0.063 | 0.607 (0.371–0.993) | **0.047** |
| Tumor diameter (cm) | 0.900 (0.858–0.945) | < 0.001 | 0.898 (0.850–0.950) | **< 0.001** |
| Tumor number |  |  |  |  |
| ≤ 3 | Reference |  | Reference |  |
| > 3 | 0.560 (0.374–0.839) | 0.005 | 0.511 (0.336–0.777) | **0.002** |
| Macrovascular invasion |  |  |  |  |
| No | Reference |  | Reference |  |
| Yes | 0.662 (0.431–1.015) | 0.059 | 1.488 (0.763–2.902) | 0.243 |
| BCLC stage |  |  |  |  |
| B | Reference |  | Reference |  |
| C | 0.588 (0.354–0.977) | 0.040 | 0.635 (0.308–1.311) | 0.219 |
| Child-Pugh |  |  |  |  |
| A | Reference |  |  |  |
| B | 0.887 (0.561–1.401) | 0.607 |  |  |
| ALT (U/L) | 1.000 (0.997–1.002) | 0.770 |  |  |
| AST (U/L) | 0.999 (0.996–1.002) | 0.646 |  |  |
| PT (s) | 0.955 (0.825–1.105) | 0.533 |  |  |
| NLR | 0.936 (0.871–1.007) | 0.075 | 0.954 (0.884–1.029) | 0.219 |
| PLR | 0.999 (0.997–1.001) | 0.327 |  |  |
| ALBI | 0.857 (0.518–1.418) | 0.549 |  |  |
| AFP level (ng/mL) |  |  |  |  |
| < 400 | Reference |  |  |  |
| ≥ 400 | 1.039 (0.697–1.549) | 0.850 |  |  |

Values in bold are statistically significant at *P* < 0.05. Abbreviations: CI, confidence interval; BMI, body mass index; ICIs, immune checkpoint inhibitors; TKIs, tyrosine kinase inhibitors; anti-VEGF, anti-vascular endothelial growth factor; HBV, hepatitis B virus; BCLC, Barcelona Clinic Liver Cancer; ALT, alanine transaminase; AST, aspartate transaminase; PT, prothrombin time; NLR, neutrophil-to-lymphocyte ratio; PLR, platelet-to-lymphocyte ratio; ALBI, albumin-bilirubin; AFP, alpha-fetoprotein.

## Table S9. Univariate and multivariate analysis of Cox regression in the training set

| Characteristics | Univariate analysis | | Multivariate analysis | |
| --- | --- | --- | --- | --- |
|  | Hazard ratio (95% CI) | *P* value | Hazard ratio (95% CI) | *P* value |
| Age (years) |  |  |  |  |
| < 60 | Reference |  | Reference |  |
| ≥ 60 | 1.283 (1.031–1.596) | 0.025 | 1.371 (1.087–1.729) | **0.008** |
| Sex |  |  |  |  |
| Female | Reference |  |  |  |
| Male | 1.117 (0.830–1.502) | 0.465 |  |  |
| BMI (kg/m^2^) | 0.953 (0.920–0.986) | 0.006 | 0.958 (0.925–0.992) | **0.017** |
| Types of ICIs |  |  |  |  |
| PD-1 | Reference |  |  |  |
| PD-L1 | 1.052 (0.669–1.654) | 0.827 |  |  |
| PD-1/CTLA-4 | 2.455 (0.912–6.610) | 0.076 |  |  |
| Treatment |  |  |  |  |
| ICIs alone | Reference |  |  |  |
| ICIs + TKIs | 0.966 (0.690–1.353) | 0.840 |  |  |
| ICIs + anti-VEGF | 0.829 (0.548–1.252) | 0.373 |  |  |
| HBV infection |  |  |  |  |
| No | Reference |  |  |  |
| Yes | 1.068 (0.761–1.498) | 0.705 |  |  |
| Cirrhosis, n (%) |  |  |  |  |
| No | Reference |  | Reference |  |
| Yes | 1.331 (1.037–1.707) | 0.025 | 1.320 (1.015–1.715) | **0.038** |
| Tumor diameter (cm) | 1.069 (1.044–1.095) | < 0.001 | 1.078 (1.047–1.110) | **< 0.001** |
| Tumor number |  |  |  |  |
| ≤ 3 | Reference |  | Reference |  |
| > 3 | 0.760 (0.611–0.945) | 0.013 | 0.817 (0.652–1.025) | 0.081 |
| Macrovascular invasion |  |  |  |  |
| No | Reference |  | Reference |  |
| Yes | 1.257 (0.995–1.588) | 0.055 | 0.930 (0.712–1.215) | 0.594 |
| BCLC stage |  |  |  |  |
| B | Reference |  |  |  |
| C | 1.235 (0.938–1.627) | 0.133 |  |  |
| Child-Pugh |  |  |  |  |
| A | Reference |  | Reference |  |
| B | 1.486 (1.160–1.904) | 0.002 | 1.238 (0.920–1.665) | 0.158 |
| ALT (U/L) | 1.001 (1.000–1.002) | 0.079 | 1.002 (1.000–1.005) | **0.036** |
| AST (U/L) | 1.001 (1.000–1.003) | 0.097 | 0.998 (0.995–1.001) | 0.274 |
| PT (s) | 1.038 (0.965–1.116) | 0.317 |  |  |
| NLR | 1.051 (1.018–1.084) | 0.002 | 1.034 (0.986–1.084) | 0.168 |
| PLR | 1.001 (1.000–1.002) | 0.018 | 1.000 (0.998–1.001) | 0.660 |
| ALBI | 1.761 (1.344–2.308) | < 0.001 | 1.222 (0.869–1.718) | 0.248 |
| AFP level (ng/mL) |  |  |  |  |
| < 400 | Reference |  |  |  |
| ≥ 400 | 0.939 (0.753–1.170) | 0.573 |  |  |

Values in bold are statistically significant at *P* < 0.05. Abbreviations: CI, confidence interval; BMI, body mass index; ICIs, immune checkpoint inhibitors; TKIs, tyrosine kinase inhibitors; anti-VEGF, anti-vascular endothelial growth factor; HBV, hepatitis B virus; BCLC, Barcelona Clinic Liver Cancer; ALT, alanine transaminase; AST, aspartate transaminase; PT, prothrombin time; NLR, neutrophil-to-lymphocyte ratio; PLR, platelet-to-lymphocyte ratio; ALBI, albumin-bilirubin; AFP, alpha-fetoprotein.

## Table S10. Correlation analyses between prediction models for early disease control in the training set

|  | ECOS-DC model | Clinical-DC model | Integrated-DC model |
| --- | --- | --- | --- |
| ECOS-DC model |  | 0.221 | 0.939 |
| Clinical-DC model | <0.001 |  | 0.504 |
| Integrated-DC model | <0.001 | <0.001 |  |

The lower half of the table is the *P* value, and the upper half of the table is the correlation coefficient.

## Table S11. Correlation analyses between prediction models for early disease control in the internal validation set

|  | ECOS-DC model | Clinical-DC model | Integrated-DC model |
| --- | --- | --- | --- |
| ECOS-DC model |  | 0.058 | 0.943 |
| Clinical-DC model | 0.418 |  | 0.313 |
| Integrated-DC model | <0.001 | <0.001 |  |

The lower half of the table is the *P* value, and the upper half of the table is the correlation coefficient.

## Table S12. Correlation analyses between prediction models for early disease control in the external test set

|  | ECOS-DC model | Clinical-DC model | Integrated-DC model |
| --- | --- | --- | --- |
| ECOS-DC model |  | 0.075 | 0.950 |
| Clinical-DC model | 0.318 |  | 0.323 |
| Integrated-DC model | <0.001 | <0.001 |  |

The lower half of the table is the *P* value, and the upper half of the table is the correlation coefficient.

## Table S13. Correlation analyses between prediction models for overall survival in the training set

|  | ECOS-OS model | Clinical-OS model | Integrated-OS model |
| --- | --- | --- | --- |
| ECOS-OS model |  | 0.273 | 0.932 |
| Clinical-OS model | <0.001 |  | 0.590 |
| Integrated-OS model | <0.001 | <0.001 |  |

The lower half of the table is the *P* value, and the upper half of the table is the correlation coefficient.

## Table S14. Correlation analyses between prediction models for overall survival in the internal validation set

|  | ECOS-OS model | Clinical-OS model | Integrated-OS model |
| --- | --- | --- | --- |
| ECOS-OS model |  | 0.144 | 0.920 |
| Clinical-OS model | 0.044 |  | 0.503 |
| Integrated-OS model | <0.001 | <0.001 |  |

The lower half of the table is the *P* value, and the upper half of the table is the correlation coefficient.

## Table S15. Correlation analyses between prediction models for overall survival in the external test set

|  | ECOS-OS model | Clinical-OS model | Integrated-OS model |
| --- | --- | --- | --- |
| ECOS-OS model |  | 0.145 | 0.971 |
| Clinical-OS model | 0.052 |  | 0.346 |
| Integrated-OS model | <0.001 | <0.001 |  |

The lower half of the table is the *P* value, and the upper half of the table is the correlation coefficient

## Table S16. Ablation analyses of ECOS-Net on early disease control prediction across the sets

| Sets | AUC of early disease control prediction (95% CI) | | | |
| --- | --- | --- | --- | --- |
|  | Habitat | 2.5D deep learning | ECOS-Net | Single-task |
| Training set | 0.742 (0.690–0.792) | 0.778 (0.734–0.824) | 0.836 (0.798–0.870) | 0.800 (0.759–0.839) |
| Internal validation set | 0.742 (0.662–0.825) | 0.769 (0.696–0.845) | 0.822 (0.758–0.883) | 0.797 (0.724–0.859) |
| External test set | 0.736 (0.647–0.814) | 0.761 (0.691–0.837) | 0.817 (0.750–0.875) | 0.795 (0.721–0.873) |

Abbreviations: AUC, area under the receiver operating characteristic curve; CI, confidence interval.

## Table S17. Ablation analyses of ECOS-Net on overall survival prediction across the sets

| Sets | C-index of overall survival prediction (95% CI) | | | |
| --- | --- | --- | --- | --- |
|  | Habitat | 2.5D deep learning | ECOS-Net | Single-task |
| Training set | 0.676 (0.648–0.704) | 0.688 (0.658–0.714) | 0.730 (0.703–0.757) | 0.704 (0.676–0.732) |
| Internal validation set | 0.661 (0.608–0.708) | 0.674 (0.623–0.726) | 0.722 (0.680–0.765) | 0.699 (0.654–0.744) |
| External test set | 0.658 (0.600–0.707) | 0.676 (0.621–0.726) | 0.720 (0.671–0.767) | 0.696 (0.651–0.741) |

Abbreviations: C-index, Harrell’s concordance index; CI, confidence interval.

## Table S18. Tumor response between groups stratified by the ECOS-DC model in all sets

| Tumor response, n (%) | Lower probability | Higher probability | *P* value |
| --- | --- | --- | --- |
| Training set (n = 456) |  |  | **< 0.001** |
| CR | 0 (0%) | 0 (0%) |  |
| PR | 13 (10.9%) | 97 (28.8%) |  |
| SD | 17 (14.3%) | 182 (54%) |  |
| PD | 89 (74.8%) | 58 (17.2%) |  |
| Internal validation set (n = 196) |  |  | **< 0.001** |
| CR | 0 (0%) | 0 (0%) |  |
| PR | 22 (23.7%) | 40 (38.8%) |  |
| SD | 27 (29%) | 53 (51.5%) |  |
| PD | 44 (47.3%) | 10 (9.7%) |  |
| External test set (n = 180) |  |  | **< 0.001** |
| CR | 0 (0%) | 0 (0%) |  |
| PR | 20 (19.8%) | 33 (41.8%) |  |
| SD | 35 (34.7%) | 40 (50.6%) |  |
| PD | 46 (45.5%) | 6 (7.6%) |  |

Values in bold are statistically significant at *P* < 0.05. Abbreviations: CR, complete response; PR, partial response; SD, stable disease; PD, progressive disease.

## Table S19. Multivariable logistic regression analyses of the ECOS-DC model and clinical factors on early disease control in the training, internal validation, and external test sets

| Characteristics | Training set  multivariate analysis | | Internal validation set  multivariate analysis | | External test set  multivariate analysis | |
| --- | --- | --- | --- | --- | --- | --- |
|  | Odds Ratio (95% CI) | *P* value | Odds Ratio (95% CI) | *P* value | Odds Ratio (95% CI) | *P* value |
| Age |  |  |  |  |  |  |
| < 60 | Reference |  | Reference |  | Reference |  |
| ≥ 60 | 0.811 (0.497–1.325) | 0.403 | 1.565 (0.730–3.352) | 0.250 | 0.855 (0.388–1.883) | 0.697 |
| Sex |  |  |  |  |  |  |
| Female | Reference |  | Reference |  | Reference |  |
| Male | 0.693 (0.359–1.337) | 0.274 | 0.566 (0.155–2.074) | 0.391 | 1.213 (0.415–3.544) | 0.724 |
| HBV infection |  |  |  |  |  |  |
| No | Reference |  | Reference |  | Reference |  |
| Yes | 1.482 (0.718–3.060) | 0.288 | 0.730 (0.201–2.651) | 0.632 | 1.797 (0.786–4.105) | 0.165 |
| Cirrhosis |  |  |  |  |  |  |
| No | Reference |  | Reference |  | Reference |  |
| Yes | 0.888 (0.501–1.573) | 0.683 | 1.078 (0.455–2.554) | 0.865 | 0.320 (0.130–0.747) | **0.008** |
| Macrovascular invasion |  |  |  |  |  |  |
| No | Reference |  | Reference |  | Reference |  |
| Yes | 1.207 (0.599–2.433) | 0.599 | 0.659 (0.200–2.167) | 0.492 | 1.280 (0.513–3.193) | 0.597 |
| BCLC stage |  |  |  |  |  |  |
| B | Reference |  | Reference |  | Reference |  |
| C | 0.553 (0.245–1.248) | 0.154 | 0.296 (0.059–1.473) | 0.137 | 0.224 (0.046–1.095) | 0.065 |
| Child-Pugh |  |  |  |  |  |  |
| A | Reference |  | Reference |  | Reference |  |
| B | 0.901 (0.509–1.595) | 0.721 | 1.032 (0.411–2.592) | 0.947 | 1.225 (0.509–2.949) | 0.651 |
| AFP level |  |  |  |  |  |  |
| < 400 | Reference |  | Reference |  | Reference |  |
| ≥ 400 | 0.903 (0.554–1.474) | 0.684 | 0.511 (0.240–1.088) | 0.082 | 0.837 (0.363–1.934) | 0.678 |
| Treatment |  |  |  |  |  |  |
| ICIs alone | Reference |  | Reference |  | Reference |  |
| ICIs + TKIs | 1.200 (0.578–2.489) | 0.625 | 1.325 (0.336–5.226) | 0.688 | 0.446 (0.040–4.980) | 0.512 |
| ICIs + anti-VEGF | 1.541 (0.623–3.812) | 0.349 | 1.741 (0.383–7.924) | 0.473 | 2.687 (0.108–66.797) | 0.547 |
| ECOS-DC model |  |  |  |  |  |  |
| Lower probability | Reference |  | Reference |  | Reference |  |
| Higher probability | 15.044 (8.916–25.384) | **< 0.001** | 9.404 (4.171–21.206) | **< 0.001** | 12.554 (4.677–33.699) | **< 0.001** |

Values in bold are statistically significant at *P* < 0.05. Abbreviations: CI, confidence interval; HBV, hepatitis B virus; BCLC, Barcelona Clinic Liver Cancer; AFP, alpha-fetoprotein; ICIs, immune checkpoint inhibitors; TKIs, tyrosine kinase inhibitors; anti-VEGF, anti-vascular endothelial growth factor.

## Table S20. Performance of the ECOS-DC model across subgroups

| Characteristics | Subgroup | Set | Metrics | | | | |
| --- | --- | --- | --- | --- | --- | --- | --- |
|  |  |  | Accuracy | Specificity | F1 score | PPV | AUC |
| Age | < 60 | TS | 0.806 | 0.643 | 0.860 | 0.837 | 0.825 |
|  | ≥ 60 | TS | 0.808 | 0.556 | 0.868 | 0.817 | 0.847 |
|  | < 60 | IVS | 0.721 | 0.824 | 0.770 | 0.897 | 0.833 |
|  | ≥ 60 | IVS | 0.671 | 0.800 | 0.745 | 0.911 | 0.805 |
|  | < 60 | ETS | 0.641 | 0.906 | 0.667 | 0.925 | 0.801 |
|  | ≥ 60 | ETS | 0.688 | 0.850 | 0.750 | 0.923 | 0.836 |
| Sex | Female | TS | 0.827 | 0.739 | 0.874 | 0.882 | 0.887 |
|  | Male | TS | 0.803 | 0.581 | 0.862 | 0.818 | 0.827 |
|  | Female | IVS | 0.727 | 1.000 | 0.800 | 1.000 | 0.986 |
|  | Male | IVS | 0.695 | 0.800 | 0.753 | 0.890 | 0.810 |
|  | Female | ETS | 0.667 | 0.750 | 0.727 | 0.857 | 0.697 |
|  | Male | ETS | 0.660 | 0.909 | 0.701 | 0.938 | 0.844 |
| BCLC stage | B | TS | 0.792 | 0.458 | 0.868 | 0.841 | 0.749 |
|  | C | TS | 0.811 | 0.634 | 0.862 | 0.824 | 0.851 |
|  | B | IVS | 0.650 | 1.000 | 0.759 | 1.000 | 0.896 |
|  | C | IVS | 0.712 | 0.800 | 0.759 | 0.877 | 0.818 |
|  | B | ETS | 0.630 | 1.000 | 0.737 | 1.000 | 0.931 |
|  | C | ETS | 0.667 | 0.878 | 0.698 | 0.908 | 0.800 |
| Child-Pugh | A | TS | 0.802 | 0.591 | 0.861 | 0.826 | 0.826 |
|  | B | TS | 0.824 | 0.649 | 0.872 | 0.833 | 0.859 |
|  | A | IVS | 0.701 | 0.833 | 0.760 | 0.913 | 0.836 |
|  | B | IVS | 0.690 | 0.750 | 0.755 | 0.870 | 0.783 |
|  | A | ETS | 0.650 | 0.919 | 0.672 | 0.933 | 0.844 |
|  | B | ETS | 0.683 | 0.800 | 0.756 | 0.912 | 0.768 |
| Cirrhosis | No | TS | 0.864 | 0.563 | 0.914 | 0.865 | 0.838 |
|  | Yes | TS | 0.785 | 0.617 | 0.842 | 0.811 | 0.830 |
|  | No | IVS | 0.660 | 0.769 | 0.735 | 0.893 | 0.796 |
|  | Yes | IVS | 0.713 | 0.829 | 0.768 | 0.907 | 0.832 |
|  | No | ETS | 0.609 | 1.000 | 0.658 | 1.000 | 0.884 |
|  | Yes | ETS | 0.694 | 0.833 | 0.734 | 0.887 | 0.798 |
| HBV infection | No | TS | 0.804 | 0.55 | 0.861 | 0.791 | 0.865 |
|  | Yes | TS | 0.808 | 0.614 | 0.864 | 0.833 | 0.830 |
|  | No | IVS | 0.550 | 0.600 | 0.640 | 0.800 | 0.787 |
|  | Yes | IVS | 0.716 | 0.837 | 0.773 | 0.914 | 0.821 |
|  | No | ETS | 0.673 | 0.842 | 0.700 | 0.875 | 0.830 |
|  | Yes | ETS | 0.656 | 0.909 | 0.707 | 0.945 | 0.804 |
| AFP level | < 400 | TS | 0.783 | 0.586 | 0.845 | 0.814 | 0.820 |
|  | ≥ 400 | TS | 0.841 | 0.633 | 0.890 | 0.846 | 0.861 |
|  | < 400 | IVS | 0.658 | 0.769 | 0.740 | 0.905 | 0.767 |
|  | ≥ 400 | IVS | 0.759 | 0.857 | 0.791 | 0.900 | 0.881 |
|  | < 400 | ETS | 0.641 | 0.905 | 0.689 | 0.939 | 0.828 |
|  | ≥ 400 | ETS | 0.676 | 0.871 | 0.718 | 0.913 | 0.809 |
| Macrovascular invasion | No | TS | 0.787 | 0.439 | 0.863 | 0.819 | 0.771 |
|  | Yes | TS | 0.817 | 0.670 | 0.864 | 0.833 | 0.860 |
|  | No | IVS | 0.672 | 0.778 | 0.774 | 0.947 | 0.822 |
|  | Yes | IVS | 0.712 | 0.822 | 0.750 | 0.877 | 0.824 |
|  | No | ETS | 0.692 | 1.000 | 0.744 | 1.000 | 0.918 |
|  | Yes | ETS | 0.643 | 0.833 | 0.682 | 0.880 | 0.765 |
| Tumor number | ≤ 3 | TS | 0.803 | 0.585 | 0.869 | 0.861 | 0.812 |
|  | > 3 | TS | 0.810 | 0.617 | 0.859 | 0.799 | 0.856 |
|  | ≤ 3 | IVS | 0.615 | 0.765 | 0.711 | 0.915 | 0.760 |
|  | > 3 | IVS | 0.771 | 0.838 | 0.806 | 0.893 | 0.877 |
|  | ≤ 3 | ETS | 0.644 | 0.850 | 0.719 | 0.932 | 0.787 |
|  | > 3 | ETS | 0.678 | 0.906 | 0.688 | 0.914 | 0.824 |
| Types of ICIs | PD-1 | TS | 0.806 | 0.607 | 0.862 | 0.823 | 0.833 |
|  | PD-L1 | TS | 0.793 | 0.500 | 0.870 | 0.870 | 0.855 |
|  | PD-1 | IVS | 0.708 | 0.837 | 0.766 | 0.914 | 0.829 |
|  | PD-L1 | IVS | 0.600 | 0.600 | 0.667 | 0.750 | 0.700 |
|  | PD-1 | ETS | 0.653 | 0.880 | 0.691 | 0.915 | 0.811 |
|  | PD-L1 | ETS | 0.750 | 1.000 | 0.824 | 1.000 | 0.900 |
| Treatment | ICIs alone | TS | 0.814 | 0.619 | 0.864 | 0.814 | 0.845 |
|  | ICIs + TKIs | TS | 0.801 | 0.600 | 0.858 | 0.822 | 0.834 |
|  | ICIs + anti-VEGF | TS | 0.829 | 0.619 | 0.885 | 0.862 | 0.856 |
|  | ICIs alone | IVS | 0.762 | 1.000 | 0.815 | 1.000 | 0.938 |
|  | ICIs + TKIs | IVS | 0.693 | 0.750 | 0.757 | 0.870 | 0.796 |
|  | ICIs + anti-VEGF | IVS | 0.686 | 1.000 | 0.732 | 1.000 | 0.885 |
|  | ICIs alone | ETS | 0.500 | 1.000 | 0.571 | 1.000 | 1.000 |
|  | ICIs + TKIs | ETS | 0.660 | 0.880 | 0.693 | 0.910 | 0.806 |
|  | ICIs + anti-VEGF | ETS | 0.733 | 1.000 | 0.833 | 1.000 | 0.857 |

Abbreviations: TS, training set; IVS, internal validation set; ETS, external test set; PPV, positive predictive value; AUC, area under the receiver operating characteristic curve; BCLC, Barcelona Clinic Liver Cancer; HBV, hepatitis B virus; AFP, alpha-fetoprotein; ICIs, immune checkpoint inhibitors; TKIs, tyrosine kinase inhibitors; anti-VEGF, anti-vascular endothelial growth factor.

## Table S21. Multivariable Cox regression analyses of the ECOS-OS model and clinical factors on progression-free survival in the training, internal validation, and external test sets

| Characteristics | Training set  multivariate analysis | | Internal validation set  multivariate analysis | | External test set  multivariate analysis | |
| --- | --- | --- | --- | --- | --- | --- |
|  | Hazard ratio (95% CI) | *P* value | Hazard ratio (95% CI) | *P* value | Hazard ratio (95% CI) | *P* value |
| Age |  |  |  |  |  |  |
| < 60 | Reference |  | Reference |  | Reference |  |
| ≥ 60 | 0.937 (0.759–1.155) | 0.541 | 0.842 (0.607–1.167) | 0.301 | 0.808 (0.577–1.133) | 0.217 |
| Sex |  |  |  |  |  |  |
| Female | Reference |  | Reference |  | Reference |  |
| Male | 1.047 (0.794–1.380) | 0.743 | 0.972 (0.564–1.676) | 0.920 | 1.301 (0.823–2.056) | 0.260 |
| HBV infection |  |  |  |  |  |  |
| No | Reference |  | Reference |  | Reference |  |
| Yes | 1.045 (0.750–1.455) | 0.796 | 0.854 (0.499–1.463) | 0.566 | 1.031 (0.730–1.457) | 0.862 |
| Cirrhosis |  |  |  |  |  |  |
| No | Reference |  | Reference |  | Reference |  |
| Yes | 1.077 (0.844–1.374) | 0.553 | 0.915 (0.628–1.333) | 0.642 | 1.235 (0.880–1.733) | 0.222 |
| Macrovascular invasion |  |  |  |  |  |  |
| No | Reference |  | Reference |  | Reference |  |
| Yes | 0.953 (0.698–1.301) | 0.760 | 1.011 (0.627–1.630) | 0.964 | 1.360 (0.919–2.012) | 0.124 |
| BCLC stage |  |  |  |  |  |  |
| B | Reference |  | Reference |  | Reference |  |
| C | 1.119 (0.784–1.598) | 0.535 | 1.324 (0.740–2.369) | 0.344 | 0.961 (0.557–1.659) | 0.887 |
| Child-Pugh |  |  |  |  |  |  |
| A | Reference |  | Reference |  | Reference |  |
| B | 1.156 (0.906–1.475) | 0.243 | 1.188 (0.784–1.799) | 0.416 | 0.865 (0.605–1.235) | 0.423 |
| AFP level |  |  |  |  |  |  |
| < 400 | Reference |  | Reference |  | Reference |  |
| ≥ 400 | 1.132 (0.914–1.401) | 0.256 | 0.961 (0.684–1.352) | 0.820 | 1.283 (0.914–1.801) | 0.149 |
| Treatment |  |  |  |  |  |  |
| ICIs alone | Reference |  | Reference |  | Reference |  |
| ICIs + TKIs | 0.809 (0.593–1.104) | 0.182 | 0.880 (0.504–1.538) | 0.654 | 0.507 (0.217–1.188) | 0.118 |
| ICIs + anti-VEGF | 0.733 (0.499–1.077) | 0.113 | 0.757 (0.396–1.448) | 0.400 | 0.252 (0.090–0.706) | **0.009** |
| ECOS-OS model |  |  |  |  |  |  |
| Lower risk | Reference |  | Reference |  | Reference |  |
| Higher risk | 2.459 (1.974–3.064) | **< 0.001** | 2.156 (1.514–3.071) | **< 0.001** | 2.285 (1.622–3.220) | **< 0.001** |

Values in bold are statistically significant at *P* < 0.05. Abbreviations: CI, confidence interval; HBV, hepatitis B virus; BCLC, Barcelona Clinic Liver Cancer; AFP, alpha-fetoprotein; ICIs, immune checkpoint inhibitors; TKIs, tyrosine kinase inhibitors; anti-VEGF, anti-vascular endothelial growth factor.

## Table S22. Performance of ECOS-Net across CT acquisition subgroups

| Characteristics | Subgroup | Set | AUC for early DC prediction (95% CI) | C-index of OS prediction (95% CI) |
| --- | --- | --- | --- | --- |
| Center | WHUH | TS | 0.828 (0.770–0.888) | 0.715 (0.663–0.762) |
|  | HCH | TS | 0.853 (0.785–0.920) | 0.723 (0.677–0.771) |
|  | APH | TS | 0.831 (0.753–0.896) | 0.731 (0.684–0.779) |
|  | WHUH | IVS | 0.815 (0.662–0.924) | 0.711 (0.637–0.783) |
|  | HCH | IVS | 0.833 (0.691–0.938) | 0.739 (0.676–0.803) |
|  | APH | IVS | 0.847 (0.725–0.948) | 0.712 (0.602–0.825) |
|  | HBCH | ETS | 0.821 (0.656–0.957) | 0.743 (0.646–0.835) |
|  | XYH | ETS | 0.845 (0.753–0.921) | 0.711 (0.634–0.789) |
|  | SAHMU | ETS | 0.833 (0.685–0.940) | 0.730 (0.633–0.830) |
|  | TFCH | ETS | 0.846 (0.564–1.000) | 0.734 (0.535–0.905) |
| Slice thickness | ≤ 1.5 mm | TS | 0.838 (0.793–0.875) | 0.737 (0.703–0.768) |
|  | > 1.5 mm | TS | 0.828 (0.735–0.915) | 0.710 (0.654–0.763) |
|  | ≤ 1.5 mm | IVS | 0.814 (0.727–0.893) | 0.741 (0.689–0.789) |
|  | > 1.5 mm | IVS | 0.813 (0.676–0.913) | 0.719 (0.640–0.797) |
|  | ≤ 1.5 mm | ETS | 0.819 (0.715–0.926) | 0.722 (0.639–0.796) |
|  | > 1.5 mm | ETS | 0.816 (0.737–0.887) | 0.716 (0.660–0.774) |
| Slice interval | 1.5 mm | TS | 0.820 (0.756–0.872) | 0.688 (0.642–0.735) |
|  | < 1.5 mm | TS | 0.850 (0.794–0.898) | 0.686 (0.642–0.725) |
|  | 1.5 mm | IVS | 0.818 (0.715–0.905) | 0.719 (0.657–0.778) |
|  | < 1.5 mm | IVS | 0.829 (0.736–0.907) | 0.711 (0.643–0.776) |
|  | 1.5 mm | ETS | 0.818 (0.745–0.879) | 0.716 (0.666–0.765) |
|  | < 1.5 mm | ETS | 0.846 (0.613–1.000) | 0.757 (0.560–0.921) |
| Tube voltage | 120 kV | TS | 0.835 (0.785–0.872) | 0.737 (0.708–0.768) |
|  | Non-120 kV | TS | 0.833 (0.751–0.900) | 0.697 (0.637–0.753) |
|  | 120 kV | IVS | 0.830 (0.732–0.902) | 0.740 (0.685–0.793) |
|  | Non-120 kV | IVS | 0.810 (0.700–0.910) | 0.708 (0.625–0.785) |
|  | 120 kV | ETS | 0.817 (0.747–0.875) | 0.720 (0.671–0.767) |
|  | Non-120 kV | ETS | / | / |
| Scanner vendor | Siemens | TS | 0.834 (0.755–0.899) | 0.711 (0.654–0.763) |
|  | GE | TS | 0.833 (0.767–0.889) | 0.678 (0.629–0.728) |
|  | Others | TS | 0.848 (0.760–0.928) | 0.686 (0.624–0.749) |
|  | Siemens | IVS | 0.833 (0.730–0.929) | 0.708 (0.618–0.791) |
|  | GE | IVS | 0.802 (0.681–0.904) | 0.696 (0.628–0.762) |
|  | Others | IVS | 0.830 (0.697–0.959) | 0.725 (0.622–0.825) |
|  | Siemens | ETS | 0.817 (0.730–0.884) | 0.729 (0.681–0.775) |
|  | GE | ETS | 0.835 (0.662–0.965) | 0.754 (0.615–0.867) |
|  | Others | ETS | / | / |

Abbreviations: TS, training set; IVS, internal validation set; ETS, external test set; AUC, area under the receiver operating characteristic curve; C-index, Harrell’s concordance index; CI, confidence interval; DC, disease control; OS, overall survival. “/” indicates that no evaluable patients were available in this subgroup.

## Table S23. Comparison of molecular characteristics between groups in the ECOS-DC model

| Characteristics | Lower probability (n = 18) | Higher probability (n = 21) | *P* value | Adj. *P* value |
| --- | --- | --- | --- | --- |
| Leukocyte Fraction, median (IQR) | 0.15 (0.09, 0.16) | 0.18 (0.12, 0.22) | 0.139 | 0.383 |
| Macrophage Regulation, median (IQR) | -0.56 (-1.03, -0.30) | -0.27 (-0.55, -0.15) | 0.041 | 0.151 |
| Lymphocyte Infiltration Signature Score, mean (SD) | -0.75 (0.96) | -0.38 (0.81) | 0.199 | 0.439 |
| IFN-gamma Response, mean (SD) | -0.65 (0.68) | -0.18 (0.67) | 0.035 | 0.151 |
| TGF-beta Response, mean (SD) | -0.71 (0.31) | -0.72 (0.31) | 0.974 | 0.974 |
| StromalScore, mean (SD) | -503.32 (369.81) | -468.86 (377.98) | 0.776 | 0.856 |
| ImmuneScore, median (IQR) | 372.62 (-178.04, 854.10) | 542.88 (173.19, 769.69) | 0.345 | 0.545 |
| ESTIMATEScore, mean (SD) | -160.14 (843.69) | 92.771 (812.12) | 0.347 | 0.545 |
| CD274, median (IQR) | 0.70 (0.38, 1.07) | 0.96 (0.80, 1.48) | 0.004 | 0.043 |
| TMB, median (IQR) | 1.08 (0.97, 1.99) | 1.46 (1.22, 1.94) | 0.414 | 0.569 |
| MSI MANTIS Score, mean (SD) | 0.32 (0.03) | 0.33 (0.03) | 0.778 | 0.856 |

Adj. *P* values were calculated using the Benjamini-Hochberg method to control the false discovery rate. Abbreviations: SD, standard deviation; IQR, interquartile range; TMB, tumor mutational burden; MSI, microsatellite instability.

## Table S24. Comparison of molecular characteristics between groups in the ECOS-OS model

| Characteristics | Lower risk (n = 17) | Higher risk (n = 22) | *P* value | Adj. *P* value |
| --- | --- | --- | --- | --- |
| Leukocyte Fraction, median (IQR) | 0.19 (0.15, 0.26) | 0.14 (0.09, 0.18) | 0.035 | 0.092 |
| Macrophage Regulation, mean (SD) | -0.25 (0.46) | -0.63 (0.46) | 0.013 | 0.073 |
| Lymphocyte Infiltration Signature Score, mean (SD) | -0.36 (0.88) | -0.70 (0.88) | 0.250 | 0.343 |
| IFN-gamma Response, mean (SD) | -0.14 (0.49) | -0.60 (0.79) | 0.042 | 0.092 |
| TGF-beta Response, mean (SD) | -0.71 (0.28) | -0.72 (0.332) | 0.951 | 0.951 |
| StromalScore, mean (SD) | -418.81 (412.01) | -535.73 (334.29) | 0.334 | 0.408 |
| ImmuneScore, median (IQR) | 542.88 (306.34, 769.69) | 248.67 (-178.04, 819.15) | 0.092 | 0.145 |
| ESTIMATEScore, mean (SD) | 240.91 (824.98) | -228.63 (784) | 0.078 | 0.143 |
| CD274, median (IQR) | 1.10 (0.93, 1.48) | 0.76 (0.46, 0.94) | 0.002 | 0.024 |
| TMB, median (IQR) | 1.52 (1.4, 2.5) | 1.08 (0.97, 1.43) | 0.026 | 0.092 |
| MSI MANTIS Score, mean (SD) | 0.332 (0.03) | 0.32 (0.04) | 0.510 | 0.561 |

Adj. *P* values were calculated using the Benjamini-Hochberg method to control the false discovery rate. Abbreviations: SD, standard deviation; IQR, interquartile range; TMB, tumor mutational burden; MSI, microsatellite instability.

## Table S25. Comparison of quantitative pathological parameters between groups in the ECOS-DC model

| Characteristics | Lower probability (n = 183) | Higher probability (n = 213) | *P* value | Adj. *P* value |
| --- | --- | --- | --- | --- |
| Density of neoplastic cells (cells/mm²), median (IQR) | 2705.90 (1646.30, 3852.40) | 2655.00 (1835.50, 3525.30) | 0.958 | 0.958 |
| Density of inflammatory cells (cells/mm²), median (IQR) | 463.22 (245.65, 835.03) | 589.81 (316.46, 1012.00) | **0.024** | 0.179 |
| Density of connective cells (cells/mm²), median (IQR) | 673.13 (416.38, 1022.20) | 708.37 (439.24, 1102.80) | 0.426 | 0.616 |
| Density of dead cells (cells/mm²), median (IQR) | 2.88 (0.46, 10.14) | 3.54 (0.68, 11.80) | 0.199 | 0.499 |
| Tumor cellularity, median (IQR) | 5.98 × 10^4^ (3.55 × 10^4^, 8.62 × 10^4^) | 5.86 × 10^4^ (4.16 × 10^4^, 7.89 × 10^4^) | 0.808 | 0.906 |
| Avg Tumor-Inflammatory Distance (µm), median (IQR) | 38.99 (29.61, 51.52) | 35.13 (27.83, 46.72) | 0.054 | 0.179 |
| Min Tumor-Inflammatory Distance (µm), median (IQR) | 2.44 (2.19, 2.72) | 2.44 (2.20, 2.64) | 0.289 | 0.578 |
| Max Tumor-Inflammatory Distance (µm), median (IQR) | 252.58 (195.10, 373.23) | 245.67 (182.68, 357.52) | 0.431 | 0.616 |
| Ripley’s K (Immune), median (IQR) | 2.88 (1.98, 4.24) | 2.84 (2.04, 4.03) | 0.815 | 0.906 |
| Tumor-Immune Colocalization, median (IQR) | 0.73 (0.57, 0.87) | 0.79 (0.63, 0.90) | **0.046** | 0.179 |

Nominal *P* values < 0.05 are shown in bold. Adj. *P* values were calculated using the Benjamini-Hochberg method to control the false discovery rate. Abbreviations: IQR, interquartile range.

## Table S26. Comparison of quantitative pathological parameters between groups in the ECOS-OS model

| Characteristics | Lower risk (n = 240) | Higher risk (n = 156) | *P* value | Adj. *P* value |
| --- | --- | --- | --- | --- |
| Density of neoplastic cells (cells/mm²), median (IQR) | 2534.20 (1699.40, 3528.40) | 2827.90 (1917.20, 3833.50) | **0.030** | 0.060 |
| Density of inflammatory cells (cells/mm²), median (IQR) | 527.27 (317.09, 951.03) | 455.22 (228.73, 887.60) | 0.058 | 0.082 |
| Density of connective cells (cells/mm²), median (IQR) | 728.20 (446.15, 1122.80) | 661.65 (404.16, 994.99) | 0.106 | 0.133 |
| Density of dead cells (cells/mm²), median (IQR) | 3.16 (0.80, 11.18) | 2.71 (0.37, 10.86) | 0.178 | 0.198 |
| Tumor cellularity, median (IQR) | 5.62 × 10⁴ (3.55 × 10⁴, 8.11 × 10⁴) | 6.28 × 10⁴ (4.30 × 10⁴, 8.94 × 10⁴) | **0.023** | 0.058 |
| Avg Tumor-Inflammatory Distance (µm), median (IQR) | 35.58 (27.55, 47.84) | 38.42 (30.51, 54.37) | **0.016** | 0.058 |
| Min Tumor-Inflammatory Distance (µm), median (IQR) | 2.38 (2.15, 2.67) | 2.51 (2.31, 2.65) | **0.039** | 0.065 |
| Max Tumor-Inflammatory Distance (µm), median (IQR) | 242.01 (179.51, 323.94) | 269.18 (195.73, 386.68) | **0.020** | 0.058 |
| Ripley’s K (Immune), median (IQR) | 2.82 (2.01, 4.06) | 2.91 (2.06, 4.25) | 0.310 | 0.310 |
| Tumor-Immune Colocalization, median (IQR) | 0.78 (0.63, 0.91) | 0.74 (0.56, 0.85) | **0.009** | 0.058 |

Nominal *P* values < 0.05 are shown in bold. Adj. *P* values were calculated using the Benjamini-Hochberg method to control the false discovery rate. Abbreviations: IQR, interquartile range.

# Supplementary Figures and Figure legends

## Figure S1. Flowchart of exclusion criteria.

Abbreviations: WHUH, Union Hospital, Tongji Medical College, Huazhong University of Science and Technology; HCH, Henan Cancer Hospital; APH, Anhui Provincial Hospital; HBCH, Hubei Cancer Hospital; XYH, Xiangya Hospital, Central South University; SAHMU, The Second Affiliated Hospital of Anhui Medical University; TFCH, Tianjin First Central Hospital; SRRSH, Sir Run Run Shaw Hospital, Zhejiang University School of Medicine.

## Figure S2. Evaluation of the optimal number of clusters.

The blue line represents the Calinski-Harabasz (CH) index, and the red line represents the Davies-Bouldin (DB) index. The vertical dashed line at k = 3 indicates the recommended optimal number of clusters.

## Figure S3. Principal component analysis (PCA) of radiomic features before and after ComBat harmonization.

(A) PCA of original radiomic features. (B) PCA of ComBat-harmonized radiomic features. Dots represent individual cases, with different colors representing different centers.

## Figure S4. Feature selection using LASSO regression.

(A) LASSO coefficient shrinkage paths. (B) Cross-validation curve showing the mean squared error (MSE) across different λ values. (C) Selected features and their coefficients at the optimal λ.

## Figure S5. Calibration curve and decision curve analyses of the ECOS-DC, Clinical-DC, and Integrated-DC models.

(A) Calibration curves of the ECOS-DC, Clinical-DC, and Integrated-DC models across the training, internal validation, and external test sets. (B) Decision curve analyses of the ECOS-DC, Clinical-DC, and Integrated-DC models across the training, internal validation, and external test sets.

## Figure S6. Heatmap of clinical features and different models’ predictions in the ICI cohort.

OS, overall survival; PFS, progression-free survival; BMI, body mass index; ICIs, immune checkpoint inhibitors; TKIs, tyrosine kinase inhibitors; anti-VEGF, anti-vascular endothelial growth factor; HBV, hepatitis B virus; BCLC, Barcelona Clinic Liver Cancer; ALT, alanine transaminase; AST, aspartate transaminase; PT, prothrombin time; NLR, neutrophil-to-lymphocyte ratio; PLR, platelet-to-lymphocyte ratio; ALBI, albumin-bilirubin; AFP, alpha-fetoprotein.

## Figure S7. Tumor response distribution in the lower and higher probability groups.

Abbreviations: PR, partial response; SD, stable disease; PD, progressive disease.

## Figure S8. Predicted probabilities in the lower and higher probability groups.

Comparison of predicted probabilities between the higher and lower probability groups in the training (A), internal validation (B), and external test sets (C). ^***^*P* < 0.001

## Figure S9. Subgroup analyses of early disease control based on the ECOS-DC model.

Odds ratios were calculated using the ECOS-DC lower probability group as the reference group. The NE indicates no outcome variation in the higher probability group (all cases achieved early disease control), and thus the odds ratio, 95% CI, and *P* value could not be reliably estimated. Abbreviations: CI, confidence interval; ICIs, immune checkpoint inhibitors; TKIs, tyrosine kinase inhibitors; anti-VEGF, anti-vascular endothelial growth factor; HBV, hepatitis B virus; BCLC, Barcelona Clinic Liver Cancer; AFP, alpha-fetoprotein.

## Figure S10. Kaplan-Meier curves of PFS in the training, internal validation, and external test sets.

(A) The Kaplan-Meier curves of the ECOS-OS model. (B) The Kaplan-Meier curves of the Clinical-OS model. (C) The Kaplan-Meier curves of the Integrated-OS model. Abbreviations: PFS, progression-free survival.

## Figure S11. Calibration curves of the survival models.

Calibration curves of the ECOS-OS, Clinical-OS, and Integrated-OS models in the training (A), internal validation (B), and external test sets (C) for 1-, 2-, and 3-year predictions.

## Figure S12. Decision curve analyses of the survival models.

Decision curve analyses of the ECOS-OS, Clinical-OS, and Integrated-OS models across the training (A), internal validation (B), and external test sets (C) for 1-, 2-, and 3-year predictions.

## Figure S13. Subgroup analyses of overall survival based on the ECOS-OS model.

Hazard ratios were calculated using the ECOS-OS lower risk group as the reference group. Abbreviations: CI, confidence interval; ICIs, immune checkpoint inhibitors; TKIs, tyrosine kinase inhibitors; anti-VEGF, anti-vascular endothelial growth factor; HBV, hepatitis B virus; BCLC, Barcelona Clinic Liver Cancer; AFP, alpha-fetoprotein.

## Figure S14. Subgroup analyses of progression-free survival based on the ECOS-OS model.

Hazard ratios were calculated using the ECOS-OS lower risk group as the reference group. The NE indicates no outcome variation in the higher risk group (all cases experienced progression or death), and thus the hazard ratio and 95% CI could not be reliably estimated. Abbreviations: CI, confidence interval; ICIs, immune checkpoint inhibitors; TKIs, tyrosine kinase inhibitors; anti-VEGF, anti-vascular endothelial growth factor; HBV, hepatitis B virus; BCLC, Barcelona Clinic Liver Cancer; AFP, alpha-fetoprotein.

## Figure S15. Subgroup analyses of overall survival based on the Integrated-OS model.

Hazard ratios were calculated using the Integrated-OS lower risk group as the reference group. Abbreviations: CI, confidence interval; ICIs, immune checkpoint inhibitors; TKIs, tyrosine kinase inhibitors; anti-VEGF, anti-vascular endothelial growth factor; HBV, hepatitis B virus; BCLC, Barcelona Clinic Liver Cancer; AFP, alpha-fetoprotein.

## Figure S16. Subgroup analyses of progression-free survival based on the Integrated-OS model.

Hazard ratios were calculated using the Integrated-OS lower risk group as the reference group. Abbreviations: CI, confidence interval; ICIs, immune checkpoint inhibitors; TKIs, tyrosine kinase inhibitors; anti-VEGF, anti-vascular endothelial growth factor; HBV, hepatitis B virus; BCLC, Barcelona Clinic Liver Cancer; AFP, alpha-fetoprotein.

## Figure S17. Biological pathway analyses of ECOS-DC and ECOS-OS models.

(A) GO and KEGG enrichment analyses of nominal DEGs downregulated in the higher probability group compared with the lower probability group in the ECOS-DC model. (B) GO and KEGG enrichment analyses of nominal DEGs upregulated in the higher risk group compared with the lower risk group in the ECOS-OS model. (C) GSEA analyses of negatively enriched pathways in the higher probability group compared with the lower probability group in the ECOS-DC model. (D) GSEA analyses of enriched pathways in the higher risk group compared with the lower risk group in the ECOS-OS model. Pathway-level significance was assessed after Benjamini-Hochberg adjustment. DEGs, differentially expressed genes; BP, biological process; CC, cellular component; MF, molecular function.

## Figure S18. Biological differences of ECOS-DC and ECOS-OS models.

(A) Comparison of immune cell abundance between lower and higher probability groups of the ECOS-DC model. (B) Comparison of immune cell abundance between lower and higher risk groups of the ECOS-OS model. (C) IFN-gamma response between lower and higher probability groups of the ECOS-DC model. (D) Leukocyte fraction, IFN-gamma response, and TMB between the lower and higher risk groups of the ECOS-OS model. Biological comparisons were assessed after Benjamini-Hochberg adjustment. Abbreviations: TMB, tumor mutational burden.

## Figure S19. Immune checkpoint gene expression and mutational landscape.

(A) Heatmap of immune checkpoint gene expression in the TCGA-LIHC cohort. (B) The waterfall plot of the top 30 mutated genes in the TCGA-LIHC cohort. One patient without mutations in the displayed genes is not shown in the waterfall plot. Abbreviations: TMB, tumor mutational burden.

## Figure S20. The cell annotation genes for 10 cell types in scRNA-seq analysis.

## Figure S21. scRNA-seq analyses between higher probability-lower risk and lower probability-higher risk groups.

(A) The left and right panels display UMAP visualizations of cells from the higher probability-lower risk and lower probability-higher risk groups, respectively. (B) GSEA analyses of biological pathways in the higher probability-lower risk group compared with the lower probability-higher risk group in some cell types. NES, normalized enrichment score.
